# Xenometabolomics reveals metabolic functional guilds unique to specific inulin subtypes in human gut microbiota cultures

**DOI:** 10.1128/msystems.01031-25

**Published:** 2025-10-22

**Authors:** Brian D. Piccolo, Ming-Hsu Chen, Renny S. Lan, Becky Moody, Tianming Yao, Ting-Yi Huang, Lindsay Pack, Sean H. Adams, Stephen R. Lindemann

**Affiliations:** 1 Arkansas Children’s Nutrition Center, Little Rock, Arkansas, USA; 2 Department of Pediatrics, University of Arkansas for Medical Sciences 155639, Little Rock, Arkansas, USA; 3 Whistler Center for Carbohydrate Research, Department of Food Science, Purdue University 462841 , West Lafayette, Indiana, USA; 4 School of Chemical and Biomedical Engineering, Nanyang Technological University 541846, Singapore, Singapore; 5 Institute of Food Science and Technology, National Taiwan University, Taipei, Taiwan; 6 Department of Surgery, University of California Davis School of Medicine 12218, Sacramento, California, USA; 7 Center for Alimentary and Metabolic Science, University of California Davis School of Medicine 12218, Sacramento, California, USA; 8 Nutrition for Transformative Healthcare Program, University of California Davis School of Medicine, Sacramento, California, USA; 9 Department of Food Science, Purdue University 462841 , West Lafayette, Indiana, USA; Pacific Northwest National Laboratory, Richland, Washington, USA

**Keywords:** dietary fiber, microbiome, microbial ecology, acetate, propionate

## Abstract

Dietary fibers promote positive health outcomes that are generally attributed to large bowel bacterial fermentation and associated bioactive metabolites. Historically, studies of the latter have focused on short-chain fatty acids such as butyrate. The gastrointestinal microbiota generate thousands of xenometabolites (microbe-derived, “non-host” metabolites). Most remain uncharacterized for composition and potential bioactivity, and little is known about the impact of fiber structure on the xenometabolome. Using LC/MS, we characterized culture supernatant metabolite profiles in human stool lineages derived from three healthy adult donors and six inulins covering a range of degrees of polymerization (DPs): chicory-derived Frutalose L90 (L90; DP ~ 3), Alfa Aesar (AA; DP ~ 5), Frutafit CLR (CLR; DP ~ 8), Frutafit IQ (IQ; DP ~ 12), Frutafit TEX (TEX; DP > 23), and the highly branched Frutafit agave inulin (AGA). Of the 1,219 LC/MS detected metabolites included in the final data analysis, concentrations of 704 were statistically significant (FDR < 0.1; Kruskal-Wallis test). Of these, 15 metabolites had a structural annotation, highlighting the large number of “unknown” xenometabolites associated with inulin substrates. Each fiber type led to distinct metabolite signatures, despite lineages displaying highly disparate microbial community structures within and across donors. This illustrates that fiber-specific metabolic functional guilds manifest despite highly diverse human gut bacteria communities. While speculative, these metabolic functional guilds could help explain why health effects of dietary fibers are prevalent across the population despite highly disparate gut microbiota patterns. The results also reinforce that fiber structures have a profound effect on the xenometabolome.

**IMPORTANCE:**

Dietary fibers can convey positive health effects, but the full suite of mechanisms and fiber type differences remains to be elaborated. Historically, most discussions have focused on the impact of fibers in promoting lower gut bacterial fermentation, leading to the generation of short-chain fatty acids (especially butyrate) and promoting growth of specific microbes. That said, health effects associated with dietary fiber are generally shared across diverse individuals harboring disparate gut microbial species, and it is increasingly appreciated that xenometabolites derived from microbial metabolism number in the thousands. In the current report, we applied metabolomics characterization to human stool cultures incubated with six structurally distinct inulin fibers. The results indicate that distinct, fiber-specific metabolite signatures manifest despite quite diverse bacterial community structures across donors. Such outcomes point to the existence of metabolic functional guilds that shape the metabolite landscape—and likely the unique bioactive characteristics—across dietary fiber types.

## INTRODUCTION

Dietary patterns, specific foods, and dietary supplements are linked to health outcomes and physiological regulation, and the gut microbiota participate in this diet-health nexus. Prevalence patterns of specific gut microbiota populations are often associated with or correlated to host health or physiological function. Examples include linkages among specific intestinal microbes or xenometabolites (microbe-derived, “non-host” metabolites) and the immune system (1–4), secretion of gut hormones (e.g., references 5, 6), kidney function (e.g., references 7, 8), amino acid metabolism (e.g., references 9–11), behavior, neuron and brain function (e.g., references 12, 13), and many other physiological systems. That specific bacteria or groups of bacteria impact physiological function is most strongly illustrated by fecal/cecal content transfer studies. For instance, donor obesity (e.g., reference 14) and depression (15, 16) phenotypes were transferred to recipients, and healthy donor feces have been effective in mitigating recurrent *Clostridioides difficile* infection (17). Although the specific mechanisms of action are not fully elaborated, molecules generated by gut microbial metabolism (hereafter, xenometabolites) can regulate host systems and can also serve as microbe-microbe signals that modify the functional and phylogenetic diversity of the microbial community.

The human gastrointestinal (GI) tract houses regionally large populations (both in numbers and biomass) of microbiota with tremendous genomic and metabolic potential, and these microbes are exposed daily to a myriad of molecules from exogenous sources (i.e., foods, diet supplements, drugs, and environmental molecules), from the host (i.e., primary bile acids, urea), and from their fellow microbes. Potential interactions among these factors underscore that a large capacity for enzymatic and non-enzymatic *de novo* xenometabolite production and the generation of co-metabolites (molecules derived from actions of both host and microbes) exists in the GI tract. Metabolomics approaches capture this metabolic landscape. Correlations among metabolite abundances and specific bacterial genera can generate insight and hypotheses as to which microbes and substrates are responsible for distinct metabolite patterns. Perhaps the most recognized examples relevant to human health are butyrogenic fermenters and microbes that convert primary to secondary bile acids. The phylogenetic dispersion of some of these traits suggests that metabolic functional guilds are more influential than specific taxon abundances. If true, this may explain why, despite the significant diversity of gut microbiota across human populations, specific phytochemicals, fibers, and other food components can yield population-wide health benefits, since they yield similar metabolite endpoints. This functional metabolic guild concept warrants further consideration and validation.

Microbes vary in their ability to attach to, degrade, and metabolize the complex substrates available in the diet, especially with respect to polysaccharides. Although some small polymers such as small oligosaccharides can be directly transported into the cytosol and be metabolized, the majority of fiber polysaccharides in the diet are either too large to be directly transported and/or are trapped in a physical matrix including plant cell walls. Thus, most dietary polysaccharides require external processing by enzymes prior to being available to microbial communities (18). In addition to transport, diversity of carbohydrate linkages in natural polysaccharides requires multiple enzymes—sometimes, encoded by multiple species—for their complete hydrolysis. Thus, even subtle differences in fiber structure may result in different metabolic outcomes. As such, they each may exert distinct impacts on health.

Here, we tested the hypothesis that fibers varying in just two structural parameters (chain length and branching) will influence their microbial processing and microbial metabolism writ large, using inulins as a model fiber class with a relatively simple range of structures. Inulins are plant carbohydrate storage polymers that are commonly added to foods as dietary fibers. Although commonly found across many plants (fruits, grains, and vegetables), inulins are industrially produced from chicory, Jerusalem artichoke, and agave. Structurally, inulins from chicory and Jerusalem artichoke are linear molecules of β(1, 2)-linked fructose with a terminal glucose residue (linked to fructose through an α(1, 2) bond) and can vary in length from three to ~60 glycosyl residues. Unlike chicory inulins, agave inulins are branched, with the addition of α(2, 6) fructose bonds leading to short side chains. As prebiotics, inulins are known to improve calcium uptake and bowel function, increase satiety and moderate postprandial glucose responses, and reduce markers of inflammation at doses as small as 5 g/day (19–22). These benefits are thought to be mediated by microbial fermentation and production of metabolites, principally short-chain fatty acids (SCFAs) (21, 23). However, the few studies that have compared inulins varying in chain length have revealed differences in physiological outcomes (reviewed in reference 24), suggesting that different inulins can drive distinct microbe-derived bioactive end products. Because inulins are composed of only two (or three, in the case of agave) bond types, theoretically only two (or three) enzymes are required for their complete degradation, and near-complete degradation can be accomplished by an extracellular inulinase (glycoside hydrolase family 32, GH32) alone. However, the size of an inulin could determine the kinetics and completeness of these enzymatic activities. Once degraded, the glucose and fructose from all inulins enter metabolic pathways through glycolysis and are fermented to species-specific metabolic end products. The broader metabolic landscape directly or indirectly impacted by microbial inulin degradation remains to be fully catalogued. Herein, we leveraged reduced-diversity human fecal bacterial cultures from different donors, grown on six specific inulins, to determine, for the first time, inulin-specific xenometabolite signatures. Furthermore, by characterizing the bacterial community structures in parallel with metabolomics analyses, we tested the hypothesis that inulin subtypes drive distinct fiber-specific metabolite profiles despite highly variable taxonomic patterns.

## MATERIALS AND METHODS

### Inulin preparations and purification

Six inulin samples, including one purchased from Alfa Aesar (#A18425, lot #10206622; Lancashire, United Kingdom) and five provided by Sensus (Roosendaal, the Netherlands), were used as fermentation substrates in this study. Among the samples from Sensus, “Frutalose L90” (lot #90.71308 002) was in syrup form and contains 90% oligofructose derived from chicory root with a degree of polymerization (DP) average of 4–5; “Frutafit CLR” (DP average of 7–9, lot #202C808727) and “Frutafit IQ” (DP average of 11–13, lot #244D802526) were produced from chicory root and in powdered form; “Frutafit TEX” (DP average > 23, lot #518B801616) contained chicory inulin powder at high purity (99.5%); and “Frutafit organic agave” inulin (lot #5FIPP16116) contained inulin powder produced from blue agave, which had a unique branching structure distinct from chicory inulins (i.e., including β2,6 fructan linkages).

Because monosaccharides (glucose and fructose) and disaccharides (sucrose) were naturally present in all inulin samples at varying concentrations and may affect microbial fermentation results, a fractionation based on size exclusion chromatography (SEC) was conducted to remove molecules from inulin samples that were less than DP2. The SEC system consisted of a single HPLC pump (LC-10AT, Shimadzu, Japan); a Rheodyne 7125 injection valve (Rheodyne Inc., Cotati, CA) fitted with a 5 mL sample loop; a refractive index detector Star 9040 (Varian Associates Inc., Walnut Creek, CA); and a glass column (XK50/60, GE Healthcare Life Science, UK) that was loaded with polyacrylamide-based Bio-Gel P-2 resin (Bio-Rad Laboratories, Hercules, CA). Inulin samples were dissolved in DI water to reach 25% (wt/vol) concentration. For every fractionation run, 5 mL of sample solution (1.25 g solids) was manually injected into the SEC system. The mobile phase (DI water) was delivered at 1.2 mL/min, and the system pressure was set at 0.9 MPa. Sample collection started 240 min after the injection, when samples reached the refractive index detector, and stopped at 475 min when DP2 molecules were detected. The collected inulin sample solutions (240–475 min) were freeze-dried to remove solvents (water) at the Purdue Advanced Lyophilization Hub (LyoHub) and milled into powder using the mortar-and-pestle method.

### Analysis of inulin molecular profiles and linkage types

Molecular profiles of inulin samples after SEC fractionation were determined using high-performance anion exchange chromatography coupled with a pulsed amperometric detector (HPAEC-PAD; AS50, Dionex Corp., Sunnyvale, CA). The HPAEC-PAD system was fitted with a CarboPac PA-100 IC column (Thermo Fisher Scientific, Waltham, MA) that was suitable for oligosaccharide analysis. Two mobile phases, A (200 mM NaOH and 120 mM sodium acetate) and B (200 mM NaOH and 600 mM sodium acetate), were employed to generate salt gradients for analyte elution. The HPAEC-PAD program was set at 100% mobile phase A from 0 to 15 min after sample injection. The concentration of mobile phase B increased progressively to 100% at 45 min, and the system shifted back to 100% mobile phase A at 50 min. Injected sample concentrations were 0.01% (wt/vol). The flow rate of the mobile phase was fixed at 1.0 mL/min.

Linkage types of the six inulin samples were determined using a partially methylated alditol acetate method tailored for fructan samples (25, 26). Briefly, 0.2 to 0.8 mg of lyophilized inulin samples were dried in a glass tube with 20 µL of methanol and nitrogen gases. Inulin molecules were methylated by CH_3_I (70 µL) in the DMSO-based solution containing NaOH (wt/vol) (125 µL). The methylated carbohydrates were extracted with dichloromethane, dried under nitrogen gas, and then hydrolyzed under mild conditions with 100 µL of 0.5 M trifluoroacetic acid at 60°C for 1 h. The reduction reaction was conducted in 2 M of NH_4_OH solution containing freshly prepared NaBD_4_ at 1 M concentration (100 µL) for 2.5 h. Subsequent additions of glacial acetic acid (23 µL) and methanol solution containing acetic acid (5% [vol/vol], 250 µL), followed by repetitive drying, were used to ensure quenching of borohydride. Acetylation was carried out using acetic anhydride (250 µL) and incubated at 100°C for 2.5 h. Then, all samples were extracted with 1 mL dichloromethane into 2 mL of aqueous solution, dried under nitrogen gas, and redissolved in acetone (500 µL). The linkage types of inulin samples were determined on a gas chromatograph–mass spectrometer (GC-MS) that was equipped with a triple-axis detector (7890A-5975C, Agilent Technologies, Santa Clara, CA) and a Supelco SP2330 column. The parameters of GC-MS were set as follows: injector temperature at 240°C, detector at 300°C, carrier gas (helium) at 1.9 m/s, and split ratio at 100 to 1, and the oven temperature program was initially 100°C for 2 min and then increased at a rate of 8°C/min until reaching 240°C (27). The acquired GC-MS spectra were matched with the database at the Complex Carbohydrate Research Center: (https://glygen.ccrc.uga.edu/ccrc/specdb/ms/pmaa/pframe.html).

### Human participants and biospecimen collection

Human stool samples were collected from three adult donors: two females aged 18 and 23 and one 25-year-old male. Inclusion criteria were: age ranging from 18 to 40 years, residing in West Lafayette or Lafayette, IN, and a normal BMI (18.5–25 kg/m^2^). Exclusion criteria included: taking antibiotics in the past three months before the experiment, heavy alcohol use, history of gastrointestinal disease or chronic metabolic disease, or consumption of probiotic products in the two weeks preceding the experiment. All donors were on their regular diets before the day of fecal collection. Each donor’s samples were collected on a separate day and processed as an individual experiment using fresh media composed of premixed components to maximize consistency across experiments. On the day of a given experiment, a fresh stool sample was received from each donor (sealed in 50 mL sterile conical tubes on ice) in the morning, immediately transferred into a Coy anaerobic chamber (Coy Laboratory Products Inc., Grass Lake, MI), and used within 30 min. The anaerobic chamber was operated under a gas mixture (90% N_2_, 5% CO_2_, and 5% H_2_) at 37°C with oxygen level controlled below 30 ppm (real-time monitored by sensor).

### Sequential passage fermentation to select inulin-specific core consortia

**Fig 1:**
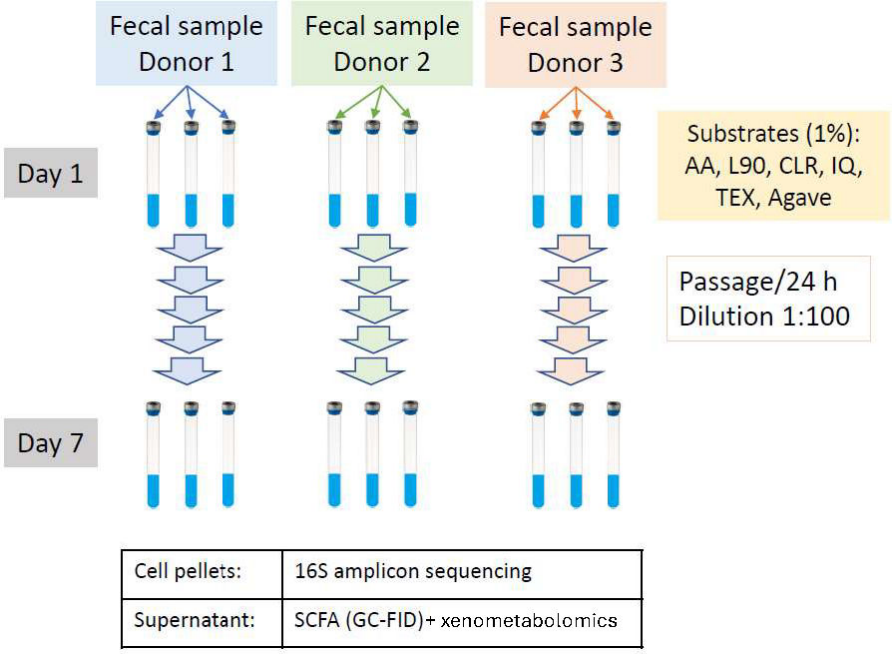
Schematic of experimental design that employed healthy adult stool cultures grown with structurally diverse inulin fibers. As described in detail in Materials and Methods, stool collections were obtained from three healthy adults and cultured for seven days with media replacement each day, generating three lineages per participant for each fiber type. Upon completion, microbiota community structures were evaluated, and an untargeted metabolomics platform was applied to post-centrifugation supernatant samples for each biospecimen. At intervals throughout the seven days of culture, short-chain fatty acids, pH, and gases were analyzed as a measure of overall metabolism and lineage bacteria viability. Abbreviations: AGA, agave inulin; AA, Alfa Aesar inulin; CLR, Frutafit CLR; IQ, Frutafit IQ; L90, Frutalose L90; Frutafit TEX.

The microbiota inoculum solution was prepared by homogenizing stool samples in a chemically defined gut mineral medium (1:20 [wt/vol] mixture) containing 40 mM sodium phosphate as the buffer (28). This medium was adjusted to an initial pH of 7.0 and was supplemented with nutrients: all 20 proteinogenic amino acids at 10 mM each, vitamins (1× ATCC MD-VS vitamin mix), and P1 trace elements (final concentrations of 34.26 mg/L H_3_BO_3_, 4.32 mg/L MnCl_2_ • 2H_2_O, 315 µg/L ZnCl_2_, 30 µg/L MoO_3_, 3 µg/L CuSO_4_ • 5H_2_O, and 12.15 µg/L CoCl_2_ • 6H_2_O). This medium is hereafter referred to as fortified buffered medium (FB) (28). The FB supports bacterial growth for controlled experimental paradigms and is not intended to mimic the dynamic and region-specific physiological conditions of the large intestine. The diluted stool slurries (20×) were filtered through four layers of cheesecloth to remove suspended solids and were used as inocula for the sequential cultures. The microbiota inocula from each of the three donors were prepared separately to avoid cross-contamination (Fig. 1).

Inulin was provided as the sole carbohydrate carbon source. Lyophilized inulin was dissolved in FB to a final inulin concentration of 1.25% (wt/vol). All inulin-containing media were sterilized by filtration through 0.22 µm polyethersulfone membranes and were maintained in the anaerobic chamber until reduced (monitored by resazurin color change). In Balch-type culture tubes (18 × 150 mm), 4 mL of inulin media was inoculated with 1 mL of microbiota inoculum, with a final inulin concentration of 1% (wt/vol). Tubes were then sealed airtight with butyl rubber stoppers and aluminum seals and incubated in a 37°C incubator, shaking at 150 rpm. Sequential passages were conducted for seven days at a passage interval of 24 h. After each 24-hour interval, Balch tubes were opened inside the anaerobic chamber for sampling. Cultures were diluted 20-fold into FB in sterile centrifuge tubes, and 1 mL of 20× diluted cultures were inoculated into 4 mL fresh inulin media. These new culture tubes were sealed and fermented for another 24 h. Daily passages were continued six times (until day 7), and the dilution rate was maintained at a ratio of 1:100 (vol/vol). All culture conditions were conducted in triplicate. Lineages of donors and inulin samples were not intermixed. At each sampling stage, the gas overpressure of each culture was recorded by piercing the rubber stopper using a 10 mL glass syringe fitted with an 18-gauge needle. Medium pH was measured using a pH meter (FEP20, Mettler Toledo, Columbus, OH). Fermented cultures were transferred into microcentrifuge tubes in 1 mL aliquots and then centrifuged at 15,871 *× g* for 10 min at 4°C. Supernatants and cell pellets were separated and immediately placed in a −80°C freezer for future microbiome and metabolite analyses.

### Community analysis using 16S rRNA gene sequencing

Genomic DNA extraction was performed on the cell pellet for microbial community analyses. DNA extraction was conducted following a modified phenol-chloroform protocol (29). Briefly, cell pellets were heated at 85°C for 5 min, followed by treatments with lysozyme solution (1 mg/mL, pH 7.2) at 37°C for 45 min and proteinase K solution (0.2 mg/mL in 10% sodium dodecyl sulfate, pH 7.2) at 56°C for 1 h. Cell lysates were transferred into a sterile screw-capped lysis tube loaded with 0.3 g zirconia beads and were homogenized using a FastPrep-24 bead beater (MP Biomedicals, Santa Ana, CA) for 10 sec. After cooling on ice, the homogenates were mixed with 0.5 homogenate volumes of phenol:chloroform:isoamyl alcohol solution (1:1:1) and centrifuged at 15,871 *× g* for 10 min. The upper aqueous phase in each lysis tube was transferred into clean 2 mL microcentrifuge tubes. Microbial DNAs were then precipitated using 50% (vol/vol) isopropanol at room temperature. After alcohol was decanted and evaporated from the pellet, the DNA was redissolved in 50 µL Tris-EDTA buffer, pH 8.0.

A library of 16S rRNA amplicons was prepared as described previously (30). DNA concentrations were normalized to 100 ng in each PCR reaction. Two primers, 515-FB (GTGYCAGCMGCCGCGGTAA) and 926R (CCGYCAATTYMTTTRAGTTT), with Illumina TruSeq adapters targeting the V4–V5 region of 16S rRNA gene were employed, yielding amplicons of ~412 nucleotides. The thermocycler conditions were set as follows: initial denaturation at 95°C for 5 min, followed by 20 amplification cycles of 98°C for 20 s, 60°C for 15 s, and 72°C for 30 s, with a final extension at 72°C for 10 min. PCR products were cleaned using the AxyPrep PCR cleanup kit (Axygen Inc., Tewksbury, MA) and then barcoded using the TruSeq indexing primers (Integrated DNA Technologies, Coralville, IA) in a second PCR reaction with five amplification cycles. After the second cleanup of barcoded PCR products using the AxyPrep PCR cleanup kit, amplicons were quantified via the Qubit dsDNA HS assay kits (Invitrogen, Carlsbad, CA). Sequencing was conducted at Purdue Genomics Core Facility using an Illumina Miseq platform (2 × 250 cycles). Resulting sequencing data were processed using mothur v1.39.3, following the Miseq SOP (https://mothur.org/wiki/miseq_sop/) with minor modifications (maximum amplicon length = 411, maximum homopolymer length = 9). Briefly, raw paired-end reads were merged and error screened before alignment to the V4–V5 region using the SILVA bacteria reference file (ver. 132). Chimeric reads were filtered using a mothur-embedded implementation of the UCHIME algorithm. Error-checked reads were then classified for taxonomy using the Ribosomal Database Project (RDP) classifier and training set (version 16, with species epithets added) (31). Operational taxonomic units (OTUs) were grouped by reads at 97% identity. Rarefaction curves were generated to calculate coverage, and the final OTU tables and ecological analyses were normalized to the smallest group size (2,854 reads). This yielded a minimum Good’s coverage of 98.3% for all samples. Ecological metrics were calculated using mothur-embedded calculators: α-diversity via species observed (sobs), Shannon, Chao, inverse Simpson (invsimpson), and Simpson’s evenness (simpsoneven) and β-diversity via the Bray–Curtis and Yue and Clayton’s theta metrics (32, 33). All sequence data can be found in the National Library of Medicine, National Center for Biotechnology Information Sequence Read Archive (SRA) database, as BioProject accession PRJNA1289892.

### Short-chain fatty acid analyses

Culture supernatants (400 µL) were mixed with 100 µL of 4-methylvaleric acid internal standard solution, which contained 50 mM 4-methylvaleric acid and 6.25 mM copper sulfate in 6% phosphoric acid. Samples mixed with standards were centrifuged at 15,871 × *g* for 10 min to remove suspended solids. Supernatants were analyzed for SCFA content via gas chromatography (GC6890, Agilent Technologies Inc., Santa Clara, CA), coupled with a flame ionization detector and a Nukol capillary GC column (SUPELCO, Bellefonte, PA), as per previously established methods (27). The operating conditions were as follows: injector temperature of 230°C, split ratio of 10 to 1, injection volume 4 µL, initial oven temperature at 100°C, increasing at a rate of 8°C per min to 200°C, helium flow rate at 0.75 mL/min, and FID temperature of 230°C. External standards included acetate, propionate, butyrate, isobutyrate, and isovalerate, premixed at a ratio of 8:2:2:1:1.

### Metabolomics analyses

Optima grade reagents, equipment, and instrumentation were obtained from Thermo Fisher Scientific (Waltham, MA) unless otherwise noted. Samples were processed in a random order across treatment groups. Bacterial culture supernatant (500 µL) was thawed on ice and vortexed 10 sec with 500 µL ice-cold methanol, followed by another 10 sec after the addition of 500 µL cold acetonitrile. Samples were kept at 4°C for 20 min and then centrifuged for 10 min at 4°C and 18,000 × *g* to precipitate proteins. The entire volume of supernatant was transferred to a clean tube, taking care to leave any protein pellet behind, and evaporated in a SpeedVac. Using a vortex (5 min) and room temperature sonicator bath (5 min), dried extracts were resuspended in 200 µL of 5% methanol containing 1,000 ng/mL each of lorazepam (Sigma Aldrich, St. Louis, MO), deuterated trans-cinnamic acid (Sigma Aldrich), and deuterated glycocholic acid (Steraloids, Newport, RI) as internal standards for real-time monitoring of assay performance (CV <10%). A quality control (QC) sample was prepared by pooling 10 µL of each reconstituted sample across all treatments. Treatment group-specific QC pooled samples were also made by mixing 10 µL of reconstituted sample within each inulin group.

Chromatography was performed on a Dionex Ultimate 3000 UHPLC, using an XSelect CSH C18 (Waters Corporation, Milford, MA) reversed phase column (2.1 × 100 mm, 2.5 µm) kept at 49°C. Metabolites were eluted using the following gradient at a flow of 0.4 mL/min: 0–2 min, 0–1% B; 2–6.5 min, 1–20% B; 6.5–11.5 min, 20–95% B; 11.5–13.5 min, 95–99% B; 13.5–16.5 min, 99–1% B; 16.5–20 min, 1% B; 20–21 min, 1–0% B; and 21–22 min, 0% B. Solvent A was 0.1% (vol/vol) formic acid in water, and solvent B was 0.1% formic acid in acetonitrile. Injection volumes were set to 5 µL for both full scan and data-dependent MS^2^ data acquisition. After conditioning the LC/MS system using the pooled QC sample, samples were injected in a randomized order by treatment group and then analyzed using a data-dependent acquisition to achieve ms^2^ spectra for compound annotation. The QC sample was injected between every 15 samples to monitor instrument drift and consistency of data acquisition. The eluted metabolites were analyzed on a Q Exactive high-resolution mass spectrometer in both positive and negative electrospray ionization (ESI±) full-scan mode, with data acquisition performed using Xcalibur 4.0 software. Nitrogen sheath, auxiliary, and sweep gas were set at 50, 13, and 3 units, respectively. Other conditions included: resolution, 70,000 FWHM; AGC target, 3 × 10^6^ ions; maximum injection time, 200 ms; scan range, 50–750 *m*/*z*; capillary temperature, 320°C; source temperature, 425°C; and spray voltages, 2.7 and 3.6 kV for negative and positive modes, respectively. Data-dependent MS^2^ spectra were generated for QC pool samples using the following conditions: resolution, 17,500 FWHM; AGC target, 1 × 10^5^ ions; maximum injection time, 50 ms; loop count, 5; isolation window, 4.0 m/z; and normalized collision energy, 30.

### Metabolomics data processing and metabolite identification

The acquired data, consisting of full MS and data-dependent MS^2^ raw files, were processed using Compound Discoverer 3.0 (Thermo) with an untargeted metabolomics workflow that included spectra selection, retention time alignment, compound detection, compound grouping across all samples, gap filling, and metabolite identification using mzCloud (online) and an “in-house” data-dependent MS^2^-fragmentation spectral database library (ddMS^2^). The latter was derived from MS^2^ fragment spectra that were derived from 420 authentic metabolite standards. Software parameters for detecting compounds were as follows: mass tolerance, 5 ppm; intensity tolerance, 30%; S/N threshold 3; minimum peak intensity, 500 k; maximum peak width, 0.4 min; and minimum scan per peak, 7. Parameters for compound groups were a mass tolerance of 5 ppm and retention time tolerance 0.15 minutes. Structurally identified metabolites were ranked according to the standard published previously (34, 35): (i) accurate mass, retention time, and MS^2^ spectra matched to in-house standard (score > 70) and (ii) accurate mass and MS^2^ spectra matched to known standard (internal or mzCloud-based; score > 70) without retention time matched. A small subset of metabolites was detected in both positive and negative modes. In this case, the data were used from the mode (for any given metabolite) that yielded the highest quantifier ion peak and lower CV% when examining QC pool runs.

Gap filling was performed across all samples using the Real Peak detection method, based on a mass tolerance of 5 ppm and S/N threshold of 1.5. Compound Discoverer (3.0) searched for missing ions with the expected *m*/*z* and RT dimensions against all detected ions while ignoring the assigned adduct type. If the node did not find a matching ion, the software attempted to detect the peak at a lower intensity threshold using the Parameterless Peak Detection (PPD) algorithm and then filled the gap with re-detected low-intensity peaks. The contrasting method uses only a peak simulation algorithm to fill the gaps with simulated chromatographic peaks. For illustration, the gap status was defined as (i) no missing adducts (no gap), (ii) at least one missing adduct (missing ions; Gap Status 2), and (ii) no adducts identified (full gap; Gap Status 3) above the intensity threshold that was used. The missing intensities for metabolites categorized in Gap Status 2 were imputed using Compound Discoverer. Metabolites with full gap status (Gap Status 3) were considered as missing or undetectable values and imputed with a zero value. Metabolomics data are available in figshare (https://doi.org/10.6084/m9.figshare.30223357).

### Data analysis and statistics

To arrive at a final, robust metabolite data set for use in statistical analysis and data modeling, a set of filtering criteria was applied. First, considering 5,239 metabolite signals (positive and negative modes), we removed 212 metabolites with full gap status (see above) across all participants. Next, 491 metabolites that were identified in ≥3 of the six culture media blanks containing a given inulin preparation were removed. Then, metabolites were filtered out from data analysis if they were not detected in at least two lineages (within a donor) and across at least two donors. These filtering steps resulted in 1,219 metabolites that were used for data analyses. Metabolite data from eight cultures with evidence of compromised growth and metabolism (see Results) were not considered in the final results. Metabolite data used for analyses are presented in Table S1. Differential abundances across all inulin groups were initially assessed using the non-parametric Kruskal-Wallis test. Results were adjusted for multiple comparisons using a false discovery rate (FDR) of 0.10 (36). Metabolites that met this criterion were then subjected to *post hoc* assessment with non-parametric relative contrast effects based on multivariate t-distribution with a Satterthwaite approximation (37). Contrasts were constructed to test differences between chicory root–derived inulins and the agave-derived inulin, in addition to all pairwise differences within chicory root–derived inulins. *Post hoc* contrast effects were considered statistically significant at FDR < 0.05. Two approaches were considered: (i) all linear structured chicory-derived inulin types compared to the branched-structure agave-derived inulin (AGA) and (ii) all pairwise comparisons among chicory inulin (without AGA).

Principal component analysis (PCA) on log-transformed metabolomics data scaled to unit variance was conducted to visualize variation associated with inulin classification across PCA components 1 and 2. Additionally, Euclidean distances of scaled, log-transformed data were subjected to agglomerative hierarchical clustering using complete linkage. The pheatmap package (38) was used to generate heatmaps of the hierarchical clustering. Associations between OTUs and metabolites across all samples and within inulins were assessed by pairwise Spearman’s correlations using the Hmisc package (39). Correlations between OTUs and metabolites were captured and adjusted for multiple comparisons using Benjamini and Hochberg FDR method. Correlations with an FDR < 0.05 were considered statistically significant. All metabolomics data were analyzed in the R Statistical Language (version 4.1.0).

## RESULTS

### Inulin fermentation metabolic outputs were highly variable across donors’ microbiota, inulin structures, and lineages

**Fig 2:**
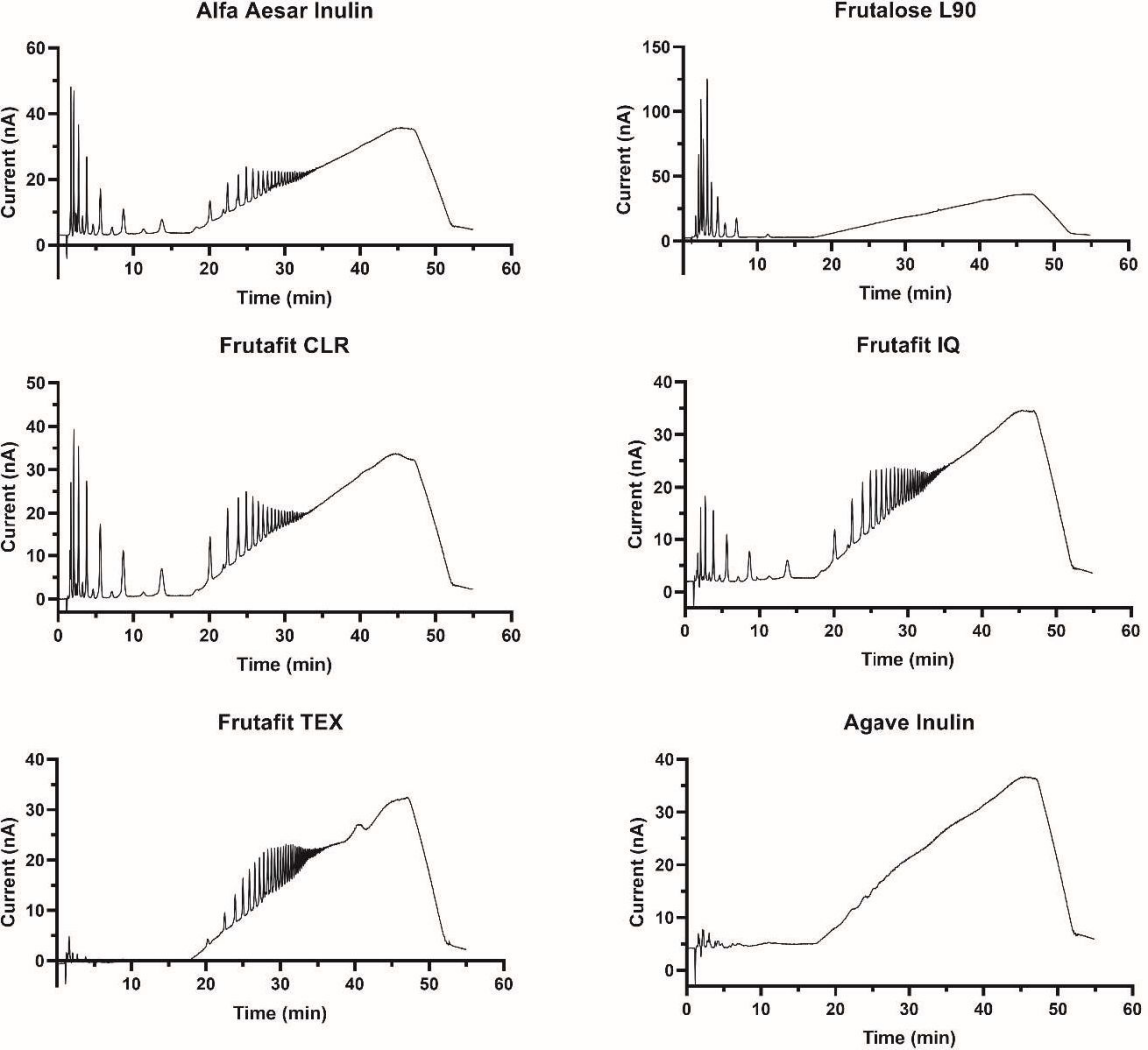
Ion chromatography results for inulin fiber types used in human stool cultures. These data confirmed that average inulin chain lengths (degree of polymerization [DP]) increased as follows: Frutalose L90 (L90; DP ~ 3) < Alfa Aesar (AA; DP ~ 5) < Frutafit CLR (CLR; DP ~ 8) < Frutafit IQ (IQ; DP ~ 12) < Frutafit TEX (TEX; DP > 23). The size distribution profile of Frutafit agave inulin (AGA) could not be determined by HPAEC-PAD, likely due to the extensive branching of this inulin type.

To determine the extent to which inulin fine structure influences the diversity and composition of fermenting communities, we performed sequential batch fermentation using inulins that varied along two structural parameters: (i) chain length and (ii) branching versus no branching. In this, we extended upon our previous work that examined only one type of inulin (Alfa Aesar), but under identical conditions (28). Ion chromatography confirmed that average inulin chain lengths (degree of polymerization [DP]) increased as follows: Frutalose L90 (L90; DP ~ 3) < Alfa Aesar (AA; DP ~ 5) < Frutafit CLR (CLR; DP ~ 8) < Frutafit IQ (IQ; DP ~ 12) < Frutafit TEX (TEX; DP > 23) (Fig. 2). Likely because of its branches, we could not acquire the size distribution profile of Frutafit agave inulin (AGA) via HPAEC-PAD. Linkage analysis data further support these inulin chain length results (Table 1). Contents of the terminal-D-glucopyranosyl residues were highest for Frutalose L90 (12.1%), indicating that Frutalose L90 had the smallest average chain length, followed by Alfa Aesar inulin (10.3%), Frutafit CLR (10.3%), Frutafit IQ (6.5%), and Frutafit TEX (4.6%). Furthermore, linkages related to branching—6-linked hexopyranosyl residue (5.9%) and 2,6-linked-D-fructopyranosyl residue (27.4%)—were detected only in Frutafit agave inulin.

**TABLE 1:** Linkage types of inulins tested in adult human stool cultures

Inulin	% linkage type (PMAA derivative)			
	T-Glu (1,5-di-O-acetyl-1-deuterio-2,3,4,6-tetra-O-methyl-D-glucitol)	2,1-Fru (1,2,5-tri-O-acetyl-1-deuterio-3,4,6-tri-O-methyl-D-frucitol)	6-Fru/6-Glu (1,5,6-tri-O-acetyl-1-deuterio-2,3,4-tri-O-methyl-D-glucitol)	2,6-Fru (1,2,5,6-tetra-O-acetyl-1-deuterio-3,4-di-O-methyl-D-frucitol)
Alfa Aesar inulin	10.3 ± 0.6	89.7 ± 0.6	N.D.^*a*^	N.D.
Frutalose L90	12.1 ± 0.5	87.9 ± 0.5	N.D.	N.D.
Frutafit CLR	10.3 ± 1.0	89.7 ± 1.0	N.D.	N.D.
Frutafit IQ	6.5 ± 0.5	93.5 ± 0.5	N.D.	N.D.
Frutafit TEX	4.6 ± 0.2	95.4 ± 0.2	N.D.	N.D.
Agave inulin	N.D.	66.7 ± 0.2	5.9 ± 0.5	27.4 ± 0.5

^*a*^ N.D., not detected.

Fecal communities varied significantly in their inulin metabolism among donors. We measured terminal acidity and gas production after each 24-hour passage. Fermentation across inulin types was generally strong across all donors after a single batch fermentation, although terminal pH and gas measurements varied significantly for some donor-inulin pairs (*P* < 0.05) (Fig. S1 and S2, time point 1). Average media pH values after the first passage were 3.90, 3.79, 3.83, 4.08, 4.26, and 4.01 (for AA, L90, CLR, IQ, TEX, and AGA inulin treatments, respectively) for Donor 1; 4.40, 4.37, 4.42, 4.45, 4.45, and 4.44 for Donor 2; and 4.00, 3.97, 4.00, 4.09, 4.09, and 4.06 for Donor 3. Gas production was generally inverse to acid production: average gas production values after the first passage from Donor 1’s microbiota were 4.83, 4.60, 4.43, 5.13, 4.87, and 5.00 mL (for AA, L90, CLR, IQ, TEX, and AGA inulin treatments, respectively); 6.97, 6.33, 5.90, 6.40, 6.03, and 7.43 mL for Donor 2’s microbiota; and 4.00, 3.77, 4.17, 4.20, 4.30, and 4.43 mL for Donor 3’s microbiota. These data suggest that (1) the distinct initial compositions of donors’ microbiota resulted in different initial fermentation kinetics and outputs on inulins and (2) inulin fermentations revealed trade-offs among acid and gas production.

**Fig 3:**
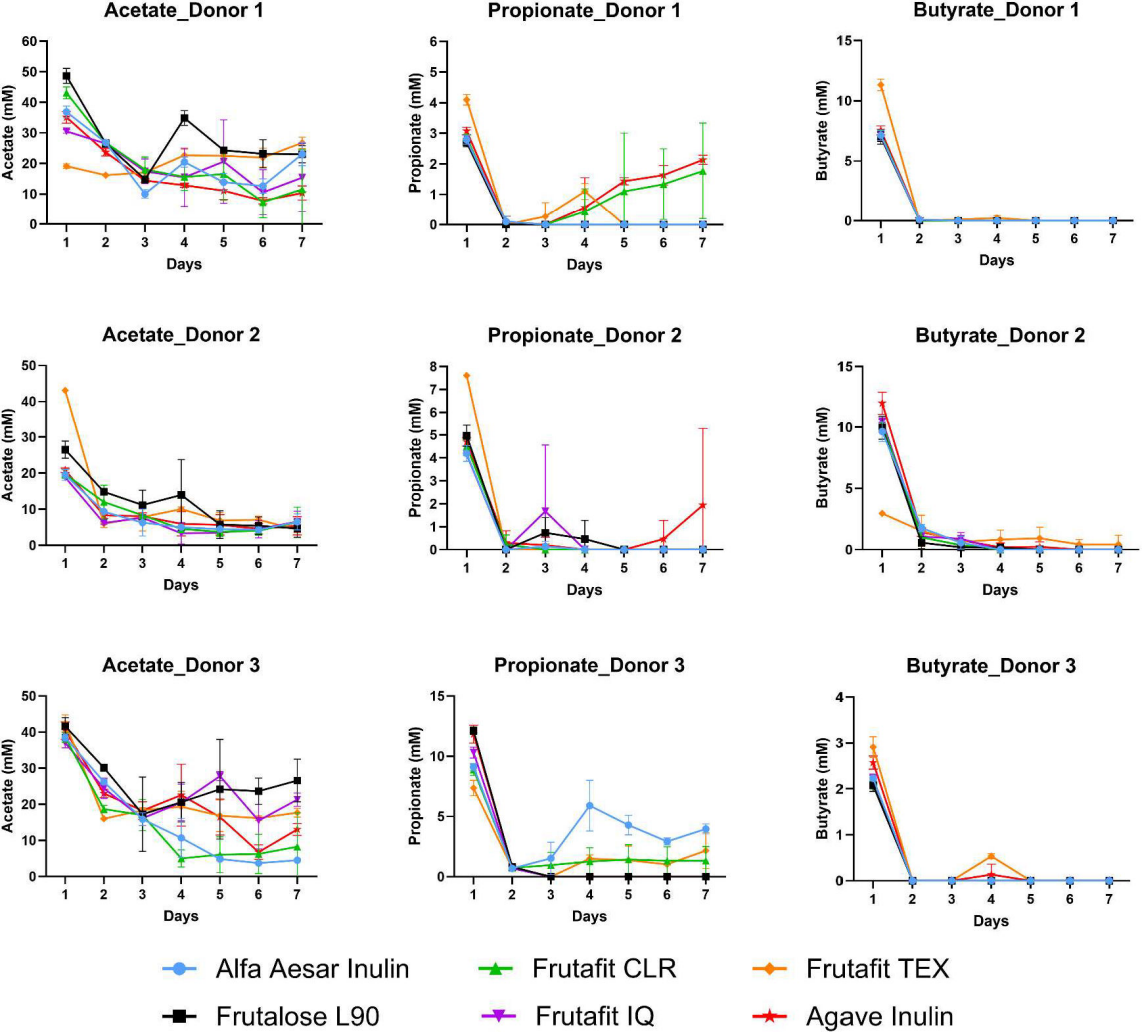
Changes in short-chain fatty acids over seven days of healthy adult stool cultures grown with various inulin fibers. Abbreviations: AGA, agave inulin; AA, Alfa Aesar inulin; CLR, Frutafit CLR; IQ, Frutafit IQ; L90, Frutalose L90; Frutafit TEX.

Initial microbiome composition interacted with inulin structure to influence the production of short-chain fatty acids (SCFAs) (Fig. 3). For Donor 1’s microbiota, acetate production after the first 24 h was roughly inverse to inulin chain length, with 19.1 mM arising in TEX fermentations and 48.7 mM in L90 fermentations. Propionate and butyrate production were nearly equivalent across inulins for Donor 1’s microbiota, except that fermentations of TEX generated substantially higher amounts of both. Conversely, Donor 2’s microbiota fermented TEX to produce substantially higher acetate and propionate but substantially lower butyrate compared with other inulins. In contrast to both, Donor 3’s microbiota fermented all inulin structures to nearly equivalent amounts of acetate but showed significant variability in propionate (with TEX lowest) and butyrate (with TEX highest). Further, the final concentrations of SCFAs, irrespective of inulin structure, varied substantially across donor inocula. Donor 1’s microbiota produced a maximum propionate concentration of 4.3 mM, Donor 2’s 7.6 mM, and Donor 3’s 12.7 mM, alongside 11.8 mM, 12.9 mM, and 3.1 mM butyrate, respectively. These data suggest that the impact of inulin structure on SCFA metabolism in fermentations is strongly determined initially by differences in donors’ microbiota composition, together with acid-gas production data implying that distinct fecal microbiota communities initially metabolize inulins to different terminal fermentation products.

### Observations of metabolically compromised lineages and definition of viable lineages for microbiome and metabolome analyses

As cultures were sequentially diluted over seven days of passaging, SCFA production dropped for all donors’ microbiota and inulin structures, alongside decreases in gas production and increases in terminal pH, but in inulin structure- and lineage-specific ways. Some lineages sustained strong metabolic output for three or four passages but seemed to become metabolically inactive at the fourth or fifth passage and did not recover. Importantly, this process appeared stochastic; often one or two lineages of a condition would fail while the remaining lineages continued to show substantial metabolism. For example, among Donor 1 fermentations of CLR, lineage 2 ceased to produce substantial acid after the fifth transfer, while lineage 1 failed to do so after the sixth. In contrast, fermentations of AA by Donor 1 microbiota demonstrated elevated terminal pH on passage 5 but recovered to be strongly acetogenic by passage 7. To account for this effect, we defined metabolic failure of a consortium as pH ≥6.5, gas production ≤1.0 mL, and acetate ≤5 mM in the terminal passage; cultures satisfying all of these conditions were determined to be failed lineages.

**Fig 4:**
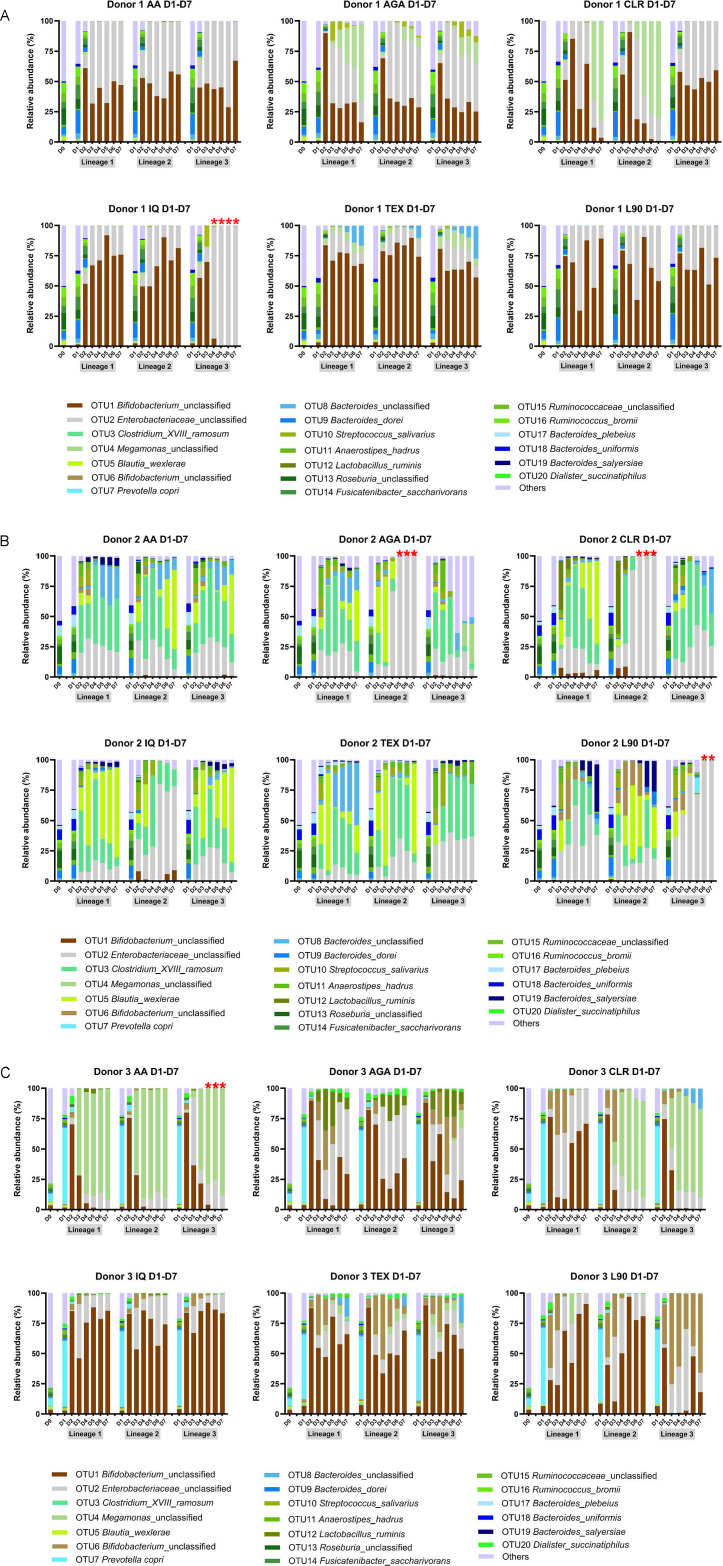
Temporal shifts in bacteria community structures in healthy adult stool cultures grown in media containing differing inulin fibers over seven days, demonstrating decreasing complexity of taxa. Left-to-right within each panel: originating stool sample, then days 1 to 7 of culture, using samples from (**A**) Donor 1, (**B**) Donor 2, and (**C**) Donor 3. Metabolomics analyses were performed on culture supernatant samples from the end of day 7. Red asterisks indicate non-viable and/or monocultures that were not used for data analyses (see Materials and Methods and Results). Abbreviations: AGA, agave inulin; AA, Alfa Aesar inulin; CLR, Frutafit CLR; IQ, Frutafit IQ; L90, Frutalose L90; Frutafit TEX.

Interestingly, failed lineages were accompanied by an almost total collapse of diversity and were often dominated by DNA from a single bacterial operational taxonomic unit (OTU; computational analogs of species) (see Fig. 4A through C). As lineages that devolved into near-monocultures of OTU2 were strongly associated with lineage failure and non-recovery, we added a taxonomic criterion: lineages exceeding 95% relative abundance of a single OTU were classified as failed lineages, serving as a second, independent criterion for lineage failure. Donor 1 lineages that failed metabolically under the above criteria were associated either with increases of *Megamonas* sp. (OTU4) or overwhelming dominance of *Enterobacteriaceae* sp. OTU2 (to the exclusion of *Bifidobacterium* sp. OTU1). Association of higher *Megamonas* sp. OTU4 prevalence with succession of failed lineages was most clear in Donor 3 communities. Where OTU4 became dominant, as in all lineages of Donor 3 cultures on AA and two on CLR, metabolic performance was weak across donors (even in cultures not meeting the formal definition of failed lineages). Failed lineages, as defined above, were excluded from analyses of culture day 7 metabolomics patterns (see below).

### Community assembly on inulin structures was stochastic across inulin structures and lineages

In contrast to the consistency we previously observed for sequential batch cultures on arabinoxylans and AA inulin (28, 40), succession on diverse inulin structures displayed stochasticity across lineages of the same substrate and donor inoculum. The compositions of fermenting microbial consortia were measured using 16S amplicon sequencing and depended on both the donors’ microbiota composition and substrate type (Fig. 4A through C). Although microbial relative abundances were not substantially altered on the first passage for Donor 1 and Donor 2 inocula, *Prevotella copri* (OTU 7) abundance increased on the first passage in Donor 3 communities across all inulin types. Donor 1 communities, in the second culture and thereafter, were dominated by *Bifidobacterium* sp. (OTU1) and *Enterobacteriaceae* sp. (OTU2) across inulin types. Successions of metabolically active Donor 1 cultures were associated with high and stable OTU1 populations. Two lineages of Donor 1 fermentations on the branched AGA maintained small but relatively stable populations of OTU4 as well as *Streptococcus salivarius* (OTU10) at passage 7. High abundances of OTU4 were associated with the lowest production of SCFAs. Donor 1 fermentations of TEX maintained sizable populations of *Bacteroides* sp. (OTU8) at the terminal passage. In contrast, microbial consortia derived from Donor 2’s microbiota were dominated by OTU2, *Clostridium*_XVIII_*ramosum* (OTU3), *Blautia wexlerae* (OTU5), *Bacteroides* spp. (OTU8, OTU9, and OTU19), and *Anaerostipes hadrus* (OTU11). Relative abundances of Donor 2 OTUs were inconsistent across lineages, and no significant differences in microbial abundance were found among inulin treatments at day 7. Notably, populations of bifidobacteria (OTU1 and *Bifidobacterium* sp. OTU6) were small and inconsistent across the succession of Donor 2 consortia. Sizable populations of OTU1 were only present in one lineage of CLR and one of IQ by passage 7, and OTU6 was only observed in intermediate fermentations of L90, being outcompeted by passage 7. Donor 3 exhibited passage 2 abundance increases in both *Bifidobacterium* spp. OTU1 and OTU6 across all inulin types; fermentations dominated by either (or, rarely, both) showed strong metabolic performance. We observed significant differences in Donor 3 cultures among inulin structures at passage 7; chiefly, consortia growing on AA and CLR were enriched with *Megamonas* (OTU4), those on AGA were especially enriched with *Enterobacteriaceae* species (OTU2), *Lactobacillus ruminus* (OTU12), and *Dialister succinatiphilus* (OTU20), while *Bacteroides* sp. (OTU8) were significantly enriched in TEX fermentations. These results suggest that (i) succession on inulins is stochastic and unstable; (ii) initial microbiota compositions strongly influence the structure and stability of fermenting consortia on different inulins varying in chain length and branching; and (iii) long-term metabolite production is tied to bifidobacterial population size.

Inulin structure did not consistently influence species richness (species observed) in communities over succession; however, in some cases, evenness parameters (Simpson’s evenness) of α-diversity were modulated by structure. As expected, the number of observed species was reduced in all treatments and lineages through sequential passages, with average evenness values bottoming out by passage 1 or 2 across donors and treatments. AGA-fermenting consortia had higher average evenness than those fermenting chicory inulins, possibly owing to AGA’s branched versus linear chicory inulin structure. Beta-diversity was quantified using Bray–Curtis dissimilarity; the influence of initial donor microbiome composition over final community composition was strong, driving donor-specific clustering.

### Inulin-associated metabolomics patterns

**Fig 5:**
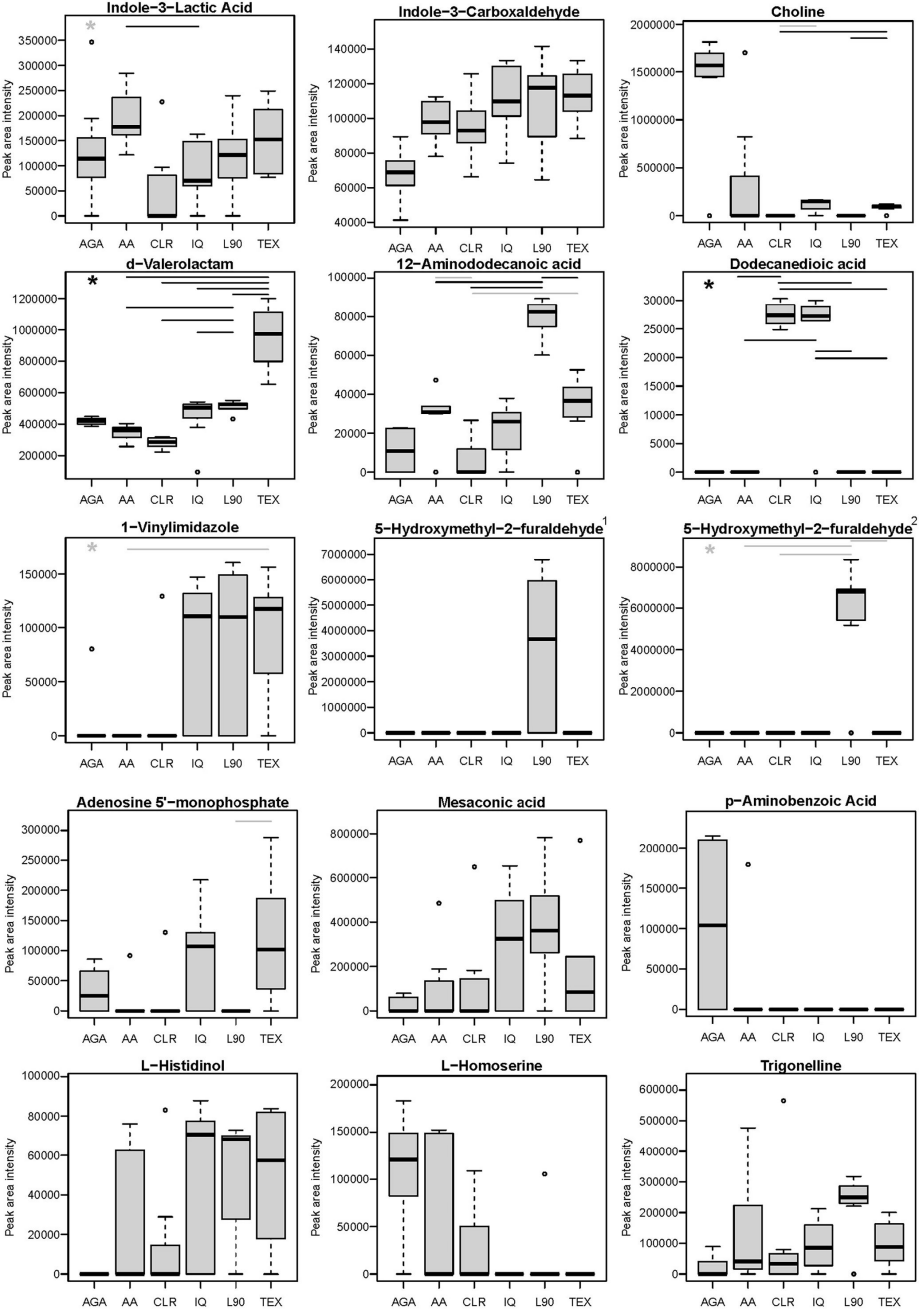
Human stool culture metabolite differences following treatment with inulins differing in structure. Metabolites were identified by LC-MS/MS from day seven passages. Duplicate metabolite annotation with superscripts “1” and “2” for 5-hydroxymethyl-2-furaldehyde represents two chemical features with RTs of approximately 0.816 and 0.964 min, respectively, and is likely isomers. Data are quantifier ion peak area intensity. An asterisk above the AGA group indicates a statistical difference between the AGA and combined ranks of inulin groups. Lines connecting one inulin structure to another indicate statistical differences of ranks between the two connecting groups. Gray asterisks and lines are raw *P*-value < 0.05. Black asterisks and lines are FDR < 0.05. Abbreviations: AGA, agave inulin; AA, Alfa Aesar inulin; CLR, Frutafit CLR; IQ, Frutafit IQ; L90, Frutalose L90; Frutafit TEX. Each inulin group consists of two to three lineages per individual donor (*n* = 3), ranging between seven and eight total lineages per group.

For each metabolite, we first tested if the concentration (semi-quantitative quantifier ion peak area) displayed a statistically significant difference when comparing across inulin types. Of 1,219 metabolites included in the final data analysis (Table S1), concentrations of 702 metabolites were statistically significant (FDR < 0.1; Kruskal-Wallis test; note that 579 metabolites were significant at FDR < 0.05) (Table S2). Of these, only 15 metabolites had a structural annotation (Fig. 5). This indicates that the specific metabolic outputs of human stool-derived bacteria are strongly impacted by the type/structure of inulin provided in the growth medium; however, the majority of these metabolites are of unknown structure. Further *post hoc* analysis of these metabolites characterized pairwise differences between inulin sub-groups and individual inulins. There were five annotated (known) metabolites (see Fig. 5 legend) and 224 non-annotated metabolites (see Table S2) that displayed an inulin-type concentration difference in at least one contrast at FDR < 0.05. Some metabolites were present for a single inulin type only (i.e., 5-hydroxymethyl-2-furaldehyde, *p*-aminobenzoic acid); others were present in some inulin conditions but absent in others (i.e., choline, L-homoserine, 1-vinylimidazole, adenosine monophosphate, dodecanedioic acid); and many were found across multiple inulins but in differing concentrations.

**Fig 6:**
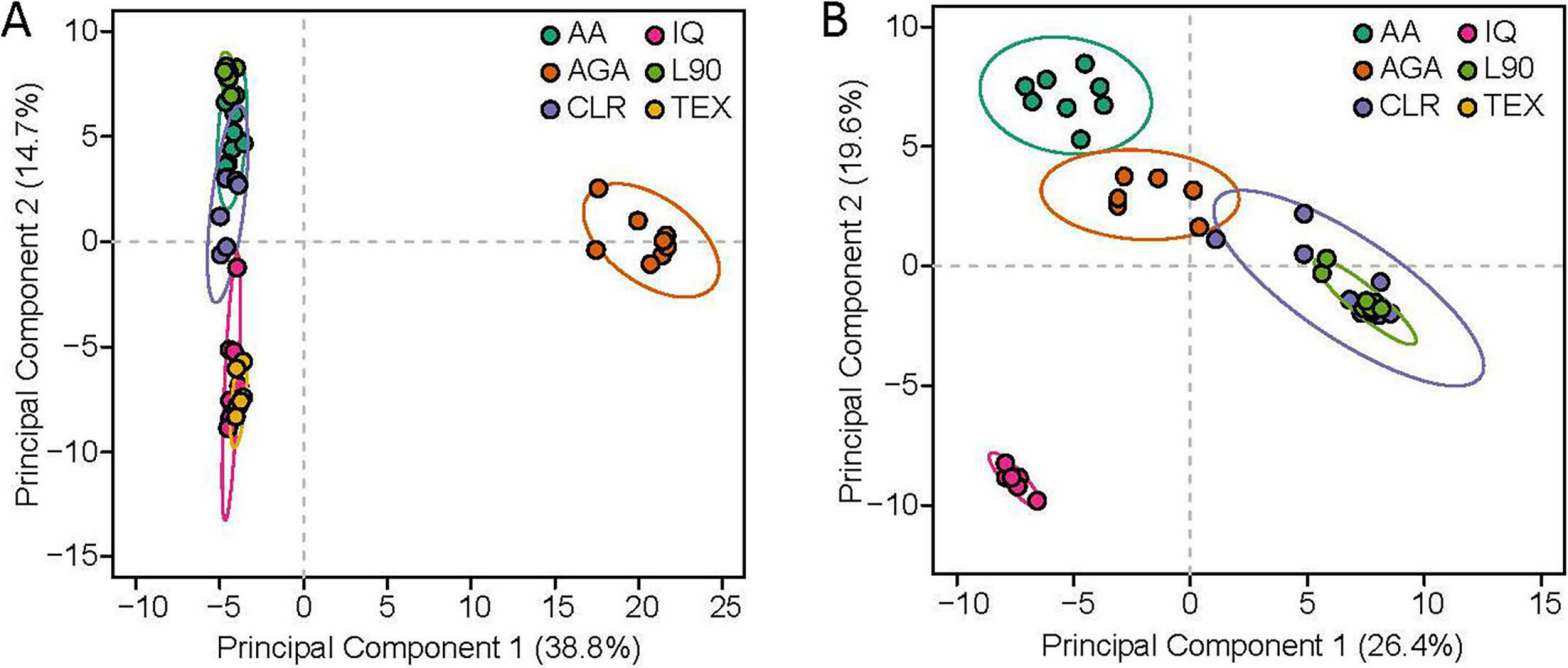
Human stool culture microbial metabolite patterns differentiate inulins with varying structures. Principal component analysis (PCA) scores plots including metabolites discriminating (**A**) all inulin structures and (**B**) chicory root-based inulin structures only. A total of 224 and 148 metabolites were utilized in A and B, respectively. Metabolites selected for inclusion were identified to have at least one pairwise difference across all inulin groups at FDR < 0.05. Metabolites were identified by LC-MS/MS from day 7 passages. Data were log-transformed and scaled to unit variance prior to analysis. Abbreviations: AGA, agave inulin; AA, Alfa Aesar inulin; CLR, Frutafit CLR; IQ, Frutafit IQ; L90, Frutalose L90; Frutafit TEX. Each inulin group consists of two to three lineages per individual donor (*n* = 3), ranging between seven and eight total lineages per group.

**Fig 7:**
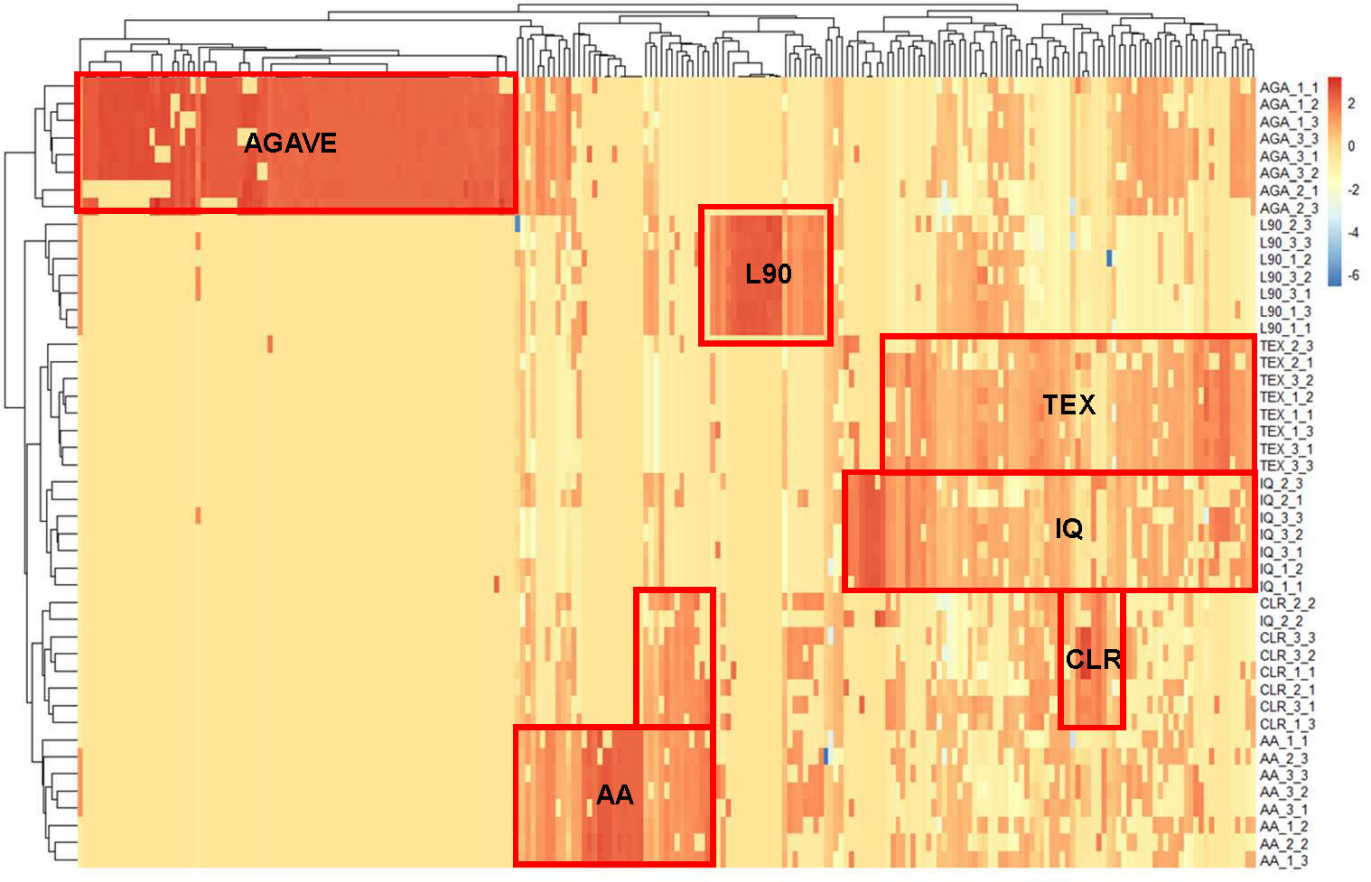
Heatmap of metabolite patterns from human stool cultures treated with inulins with varying structures. Differences are visualized across each inulin structure and lineage using a heatmap of 224 metabolites. Metabolites selected for inclusion were identified to have at least one pairwise difference across all inulin groups at FDR < 0.05. Metabolites were identified by LC-MS/MS from day 7 passages. Data were log-transformed and scaled to unit variance prior to analysis. Agglomerative hierarchical clustering of samples and metabolites in the heatmap was conducted using complete linkages on Euclidean distances. Abbreviations: AGA, agave inulin; AA, Alfa Aesar inulin; CLR, Frutafit CLR; IQ, Frutafit IQ; L90, Frutalose L90; Frutafit TEX. Each inulin group consists of two to three lineages per individual donor (*n* = 3), ranging between seven and eight total lineages per group.

To further define inulin-specific variation in metabolites, principal component analysis (PCA) was conducted on the 229 metabolites (five annotated, 224 non-annotated) noted above that displayed significant differences at FDR < 0.05 in *post hoc* contrasts (a PCA using all 1219 metabolites regardless of statistical significance is illustrated in Fig. S3). Component 1 explained >38% of the variation and visually indicated that these metabolites could readily discriminate the AGA-derived inulin–treated samples from all chicory-derived inulins (Fig. 6A). The specific metabolites that drive this outcome can be seen in their PCA model loadings values (Table S3). To refine inulin type-discriminating metabolites considering the distinct AGA-associated pattern, a second PCA was generated without AGA data (Fig. 6B). PCAs, including all inulin groups and only chicory inulins, were developed. The latter PCA (i.e., only chicory inulins) was conducted by removing AGA samples prior to data scaling/centering. After removing AGA-specific metabolites, this PCA was conducted with 148 metabolites and demonstrated four separate clusters that clearly define the TEX/IQ, AA, CLR, and L90 treatments across the first two components, explaining 46% of the variation in the data. PCA loading values for the first two components can be seen in Table S4. The interpretations derived from these PCAs are bolstered by visual examination of a heatmap depicting scaled abundances of the 229 metabolites used for PCA across all inulin, donors, and lineages (Fig. 7). Results from the heatmap indicate that AGA metabolite patterns are distinct from the other treatments, similar to results from the PCA. Consistent with PCA without AGA samples, the heatmap results show that the L90 treatment led to a unique metabolite signature; TEX and IQ appeared to be more similar to one another, AA was distinct, and CLR patterns shared some features with AA and TEX/IQ. Individual metabolite data are provided in the Supplemental Material.

### Associations between stool bacteria cultures and metabolite patterns

**Fig 8:**
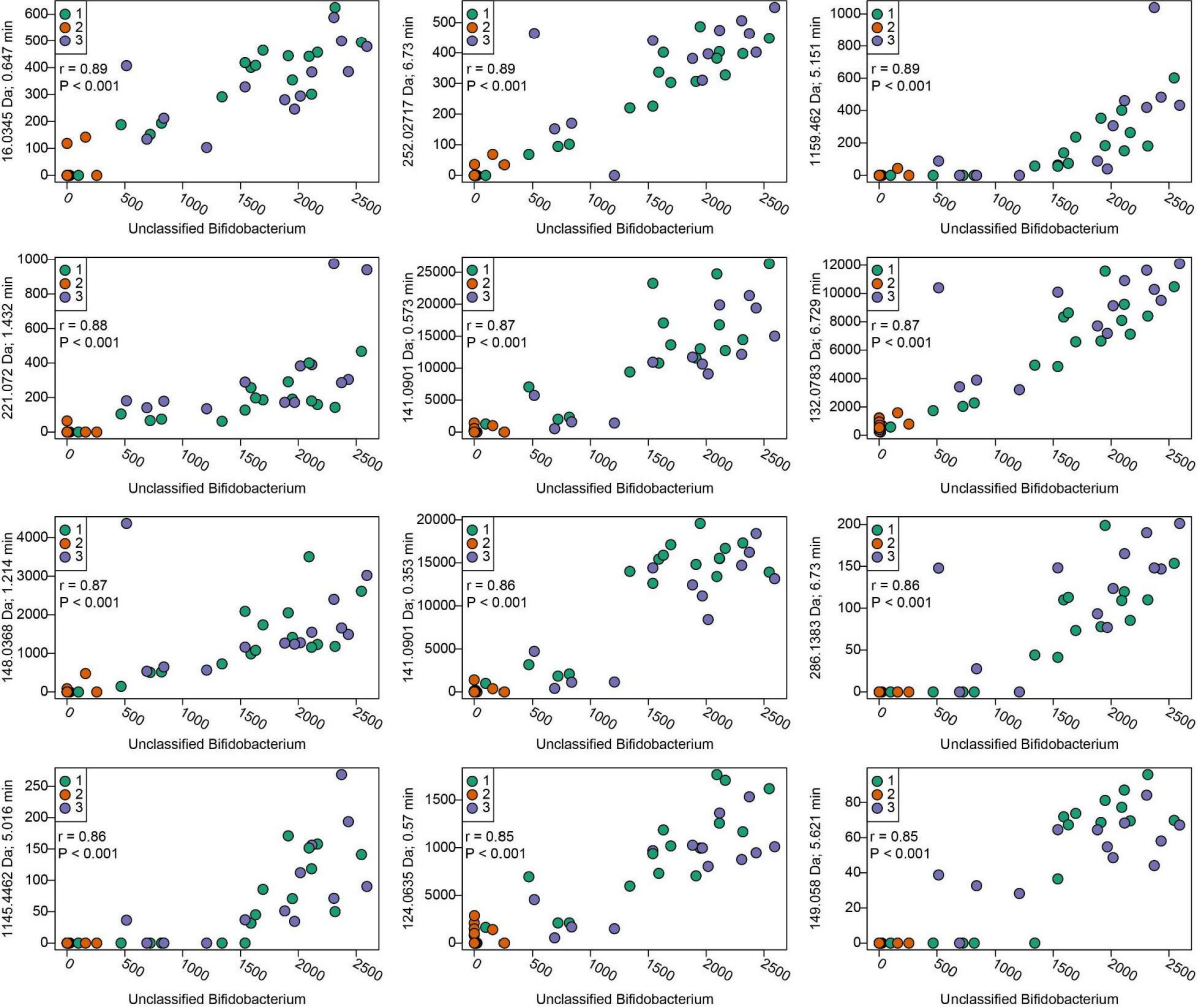
The top 12 Spearman’s correlations between *Bifidobacterium* sp. and metabolites that significantly differed in at least one binary comparison of inulins in human stool cultures. Correlations are ranked based on the largest absolute Spearman’s correlation coefficient (ρ), and are derived from seven or eight lineages per donor, per inulin type. The circle colors indicate donor. The identities of the correlated metabolites remain unknown and thus are indicated with “ID” numbers in lieu of chemical annotations. For the sake of brevity, metabolite peak area intensities are displayed as peak area intensities divided by 100.

A notable observation was that despite disparate bacterial community structures across donors and lineages (Fig. 4A through C), the metabolite profiles associated with specific inulins were distinct (see Fig. 5 to 7). This suggests that inulin type-associated metabolite patterns result from metabolic functional guilds rather than solely on specific microbial taxa. To evaluate this idea in greater detail, an exhaustive analysis was conducted to determine correlations between all 1,219 metabolites initially used for statistical analyses and the dominant bacterial OTUs characterized in the lineages. For the purposes of this analysis, significant OTUs were defined as those with >0.1% relative abundance in more than one sample. The results are quite complex and variable across the OTUs and metabolites, and despite statistically significant Spearman’s rank correlation coefficients in some cases, many correlations were not robust upon visual inspection. For instance, relationships between metabolite and OTU abundances were not always clear due to a limited range of values for a given variable (i.e., multiple zeros for a specific taxon abundance). Interpretations of correlations must be tempered by the potential for Type I statistical error in correlation analyses involving such a large number of metabolites and OTUs. That said, we did observe that for OTU1 (*Bifidobacterium* sp.), there were several statistically significant positive correlations to non-annotated metabolites, which appeared to have a linear relationship over a range of OTU abundance values (Fig. 8). Essentially, the same results were observed when we accounted for the compositional nature of OTU measures (and thus, potential for negative correlation bias) by using Spearman’s correlations with metabolite intensities and centered-log ratio transformed OTUs (Fig. S4). In summary, there were a very limited number of robust correlations between specific OTUs and specific metabolites, further supporting the concept that metabolic functional guilds drove the fiber-specific metabolite signatures that were observed.

## DISCUSSION

Epidemiology studies consistently indicate that adequate intake of dietary fiber is associated with positive health outcomes and lower risk for a variety of conditions including certain cancers, cardiovascular disease, type 2 diabetes, non-alcoholic fatty liver disease (NAFLD), and obesity (reviewed in references 41–47). Based on this body of work and in relationships between low fiber intakes and higher risk for cardiovascular disease, the 2020–2025 Dietary Guidelines for Americans considers fiber as a nutrient of public concern and recommends intakes of at least 14 g/kcal and up to 30 + g/day in adults, depending on factors such as age, sex, and daily calorie needs (48). Less well defined are the type(s) of fiber that should be consumed and fiber-specific mechanisms of action. Discussions of the latter typically focus on promoting growth of specific gut microbes (e.g., bifidobacteria, lactobacilli) and fiber as a substrate for bacterial production of xenometabolites, such as short-chain fatty acids (SCFAs). For instance, in the colon, butyrate oxidation promotes a drop in *p*O_2_ (oxygen partial pressure) that supports gut barrier function and the maintenance of a normal anaerobic luminal environment. Microbe-derived butyrate has been shown to activate the hypoxia-inducible factor signaling pathway, which has been linked to the upregulation of tight junctions and other factors associated with barrier function (49, 50). In addition, these conditions promote a normal anaerobic environment that limits overgrowth of oxygen-tolerant, respiring enterobacterial species such as *Escherichia coli* or *Klebsiella pneumoniae* (51). SCFAs can also signal through one or more receptors (e.g., GPR41 [FFAR3], GPR109A, GPR43 [FFAR2]) in a SCFA-specific manner to regulate enteroendocrine cell and islet hormone release, gut inflammation, and liver and adipocyte phenotypes (52, 53).

Despite a substantial literature regarding SCFAs and several other xenometabolites known to impact host physiology, there is a need for more comprehensive exploration of the thousands of metabolites reflective of the metabolic capacity and complex biochemistry of gut microbes and how metabolism responds to differing fiber types. Regarding the latter, naturally occurring food fibers, fibers provided as supplements, and fibers added as food ingredients can differ significantly in structure (e.g., branching, chemical composition, chemical bonds); the latter would likely impact the nature of microbial metabolisms of the fibers. Herein, we leveraged an untargeted metabolomics platform to analyze human stool-derived bacterial cultures grown with structurally-diverse inulins. The studies revealed novel inulin type-specific metabolite signatures and remarkable evidence for fiber-specific metabolic functional guilds within otherwise complex and highly variable bacteria communities.

Given the widespread fermentability of inulin substrates by many microbial species (54), we anticipated stable, if relatively stochastic, community succession over time (passages) compared to more deterministic succession we have observed on more complex polysaccharides (28, 40). In contrast, we observed surprisingly inconsistent community assembly and stability in cultures grown with inulins. Furthermore, some lineages devolved in diversity and metabolic outputs (and, often, both), suggesting a significant reduction in metabolic performance (defined with respect to pH reduction, gas, and acetate production) despite inoculation into fresh media. We observed that within any specific inulin treatment, the bacterial community composition, stability, and overall metabolic performance were donor-dependent. Observationally, these outcomes appeared to be more consistent in cultures provided longer-chain inulins (IQ, TEX), except in cultures inoculated with Donor 2 microbiota. Metabolic performance of consortia was highly correlated with the size of bifidobacterial populations in each lineage; failed lineages were rare where sizable populations of bifidobacteria were observed. Given that *Megamonas* spp. more commonly established in Donor 2-derived lineages and sizeable populations of bifidobacteria were rare, this may have resulted in relatively high failure rates of these cultures compared to that of other donors’ microbiota. Donor 2 cultures that exhibited relatively stronger metabolic performance also commonly displayed late abundance increases of OTU0005 *Blautia wexlerae*, although this did not rescue metabolic performance in all cases. The reasons underlying the diverse community compositions within inulin treatments are not clear, but may be due to passage times, which were kept long in this study to account for the relatively slower fermentation rates of longer-chain inulins. Alternatively, stress tolerance differences, like acid stability or phage induction, or differences in hydrogen gas accumulation may have been in play.

The most remarkable finding from these experiments is that despite highly variable community structures across donors, there were inulin-specific metabolite patterns that were shared across donor lineages. Although we had initially expected inulin chain length would select for distinct organisms and, therefore, different metabolite patterns, we instead found stronger evidence that specific fibers drive unique metabolic signatures despite highly diverse microbial community structures across donors. This is consistent with data suggesting a conserved metagenome (e.g., references 55, 56), especially with respect to the machinery to degrade simple fibers like inulins, and highlights the presence of metabolic functional guilds, in which similar biochemical machineries function across diverse taxa when exposed to a specific fiber type. This could emerge from individual, distinct microbes that share similar biochemical pathways and/or multiple community members that each contribute one or more components of a complex metabolic transformation of substrate. In this sense, a metabolic functional guild can be defined as a group of taxonomically distinct organisms harboring a shared functional property in terms of metabolism. The idea of guilds is by no means new to microbial ecology (for examples among many, see references 57–60); however, this principle tends to be applied at very general levels (e.g., “methanogens,” “sulfate reducers,” “aerobic heterotrophs”). Wu et al. highlighted the potential importance of the guild concept when applied to the associations between host phenotypes, gut microbiota communities, and metagenomes (61). Here, we extend this concept to gut microbial metabolic functional guilds as manifested in fiber-specific metabolite signatures. Perhaps the strongest evidence in support of this concept was the observation that very few statistically significant and plausible correlations were observed when comparing individual bacterial species (OTUs) to individual metabolites. It is well known that depending on enterotype, diet, and other factors, one’s GI tract can harbor differing abundances of microbes producing a particular metabolite: i.e., butyrate producers, lactic acid bacteria, or methanogens. Recently, the idea of a succinotype (presence of succinate-metabolizing gut-derived bacteria) was considered (62). However, by interrogating hundreds of metabolites, the current results illustrate how a functional metabolic guild can be marked by changes in multiple metabolic pathways, well beyond a single metabolite or metabolite class, and that there are xenometabolite patterns that predominate depending on inulin type. It is possible that these patterns emerge, in part, from differences in biophysical and biochemical influences. For instance, although the compositions of inulins varying in chain length are very similar with respect to glycosyl residue composition, the rate of importation into cells and the average size of imported oligosaccharides is likely to vary substantially with chain length (that is, longer chains likely relating to slower flux and larger oligosaccharides, as their utilization depends, in part, upon hydrolysis by extracellular enzymes).

Although more studies are needed to understand which metabolites regulate host physiology and microbe-microbe cross-talk, these findings support the concept that associations between specific fibers and health emanate from a multitude of bioactive metabolites that are substantially driven by fiber structure. It is intriguing to consider that the presence of functional metabolic guilds explains why dietary fibers promote health and function in most people (63) despite quite disparate and dynamic gut microbial community structures across the population (see, e.g., references 64, 65). One interesting extension of this concept is the observation that inulin(s) and fructooligosaccharides commonly promote “beneficial” bifidobacteria and lactobacilli *in vitro* (66, 67), but expansions of these microbiota are inconsistently observed in human feeding trials (68, 69). We propose that, despite variability in gut microbial community structures metabolizing a specific fiber, a harmonized fiber-specific xenometabolome profile may result in most people. This hypothesis requires testing in controlled feeding trials that interrogate fiber-associated changes in the gastrointestinal tract xenometabolome across a diverse population. Further knowledge in this area of fiber type-dependent differential metabolism could have profound implications for refinement of dietary guidelines for specific fiber recommendations, pre- and probiotic synergy, and for the design of personalized nutrition strategies.

Historically, most discussion of dietary fiber and gut microbe metabolism has centered on enhanced production of SCFAs and especially butyrate. Recent reports have highlighted that the provision of fibers such as inulin can alter hundreds, if not thousands, of xenometabolites. For example, in a study comparing the fecal metabolome from mice fed 10% chicory inulin for 16 weeks vs zero inulin controls, ~600 significantly increased metabolites were detected in positive mode and ~600 in negative mode when using an UHPLC-MS-TOF analysis platform (70). Acute studies using oral ^13^C-inulin (chicory) administration in mice demonstrated isotope enrichment in a broad variety of metabolite chemical classes in cecal contents within hours (71). Net increases in scores of metabolites across chemical classes were also observed in single-strain cultures of six commensal bacteria relevant to the human gut microbiome, grown with agave-derived inulin (72). These reports, and the data herein, bolster the principle that fibers drive profound changes in multi-pathway microbial metabolism. Thus, we submit that considering the physiological effects of fibers through the lens of SCFA production is too limited.

A full evaluation to determine the catalog of inulin fiber-associated metabolites that impact host physiology and health remains a subject for future research. Nevertheless, several annotated metabolites that were altered in at least one treatment herein are notable, and several are mentioned here. We observed, for instance, that several inulin treatments (and especially AGA) led to net increases in media choline levels. Interestingly, choline was higher in feces of mice consuming chicory-derived inulin after receiving human fecal material transfer (73), and it was a major isotope-labeled cecal metabolite in mice acutely challenged with ^13^C-inulin, as well as in human stool cultures provided with ^13^C-inulin substrate (71). The latter investigators postulated that the generation of choline was via methionine-derived ^13^C-methyl group transfer to ethanolamine. The role of choline is myriad in metabolic pathways, and certain inulins and other fibers can stimulate the *de novo* synthesis of amino acids, thereby contributing to intestinal and body-wide choline status. We also noted that indole derivatives, such as indole-3-lactic acid and indole-3-carboxaldehyde, were increased across the inulin treatments, qualitatively consistent with increased indole derivatives in mouse cecum or gut commensal cultures provided with inulin (70, 72). Indole is a tryptophan-derived xenometabolite with many metabolic fates, and some indole derivatives are generally considered to be anti-inflammatory and to promote tissue repair and protection primarily through activation of the aryl hydrocarbon receptor (AHR), a ligand-activated transcription factor (see, e.g., reference 74). Finally, Bedu-Ferrari reported generation of mesaconic acid (aka 2-methylfumaric acid) in cultures of commensals grown with inulin (72), similar to our human stool culture results. Endogenous mesaconic acid can be derived from itaconic acid, and these have been implicated as inhibitors of inflammatory pathways in macrophages (see reference 75). Recently, mesaconic acid was also described as a bacterial metabolite (in *Bacillus subtilis* R0179) with antibiotic properties against *Porphyromonas gingivalis* ATCC 33277 (76). Taken together, our results and those from the extant literature support the postulate that the physiological effects of dietary fibers—including inulins—emanate from bacterial production of a wide variety of metabolites *in situ*. The full suite of fiber-associated bioactive xenometabolites remains to be discovered, but this knowledge is critical to pursue to unlock the mechanisms that underpin the health effects of dietary fiber.

In summary, our study reveals that even dissimilar microbial community structures possess functional redundancies for metabolism of different inulins—here, varying in chain lengths—to yield similar metabolic outcomes within a fiber type. The results indicate that (i) metabolic outcomes of fiber fermentation are quite sensitive to small differences in degree of polymerization despite otherwise similar carbohydrate linkage structure, and (ii) fiber type-associated metabolite signatures can arise due to the presence of metabolic functional guilds in microbiota derived from the human gut. By leveraging stool cultures, we are able to convincingly demonstrate that these fiber-type xenometabolite patterns arise from bacterial metabolism, not from interactions with the host or gut luminal environment. We acknowledge that our study conditions are optimized to identify fiber-derived metabolites in defined environments, whereas *in vivo* differences in host processes (e.g., diet, nutrient and metabolite uptake, disease states) or physicochemical parameters (e.g., pH, redox state) across individuals may substantially alter the fiber-microbiota-xenometabolite pattern associations. Further, *in vivo* fiber fermentation occurs in dense and diverse colonic communities rather than those selected over sequential transfers, which may alter the relationship between chain length and metabolic outcome observed herein across fecal inocula. Another potential limitation of our study is that a highly conservative approach to metabolite filtering was applied to arrive at the final set of variables for statistical modeling, which may have reduced our ability to detect the entire suite of inulin-specific metabolites. Despite these interpretive caveats, our results support the concept that taxonomically diverse metabolic functional guilds of human gut-derived microbiota generate shared metabolite profiles in response to structurally diverse fibers. The nature of metabolic functional guilds awaits further elaboration in diverse populations of healthy and diseased humans, through evaluation of stool and bioregional xenometabolome following consumption of specific inulins and other fibers.

## Data Availability

All sequence data can be found in the National Library of Medicine, National Center for Biotechnology Information Sequence Read Archive (SRA) database, as BioProject accession PRJNA1289892. Metabolomics data may be found in figshare (https://doi.org/10.6084/m9.figshare.30223357).

## SUPPLEMENTAL MATERIAL

Fig. S1 msystems.01031-25-s0001.tif pH changes over time.

Fig. S2 msystems.01031-25-s0002.tif Gas production over time.

Fig. S3 msystems.01031-25-s0003.tif PCA using all metabolites.

Fig. S4 msystems.01031-25-s0004.tif Correlations using transformed OTUs.

Legends msystems.01031-25-s0005.docx Legends for Fig. S1 to S4

Supplemental Tables msystems.01031-25-s0006.xlsx Tables S1-S4.

ASM does not own the copyrights to Supplemental Material that may be linked to, or accessed through, an article. The authors have granted ASM a non-exclusive, world-wide license to publish the Supplemental Material files. Please contact the corresponding author directly for reuse.
