# Supplementary figures and images for: Xenometabolomics reveals metabolic functional guilds unique to specific inulin subtypes in human gut microbiota cultures
Source: mSystems. 2025 Oct 22;10(11):e01031-25. doi: 10.1128/msystems.01031-25 (PMC12625710; doi:10.1128/msystems.01031-25)

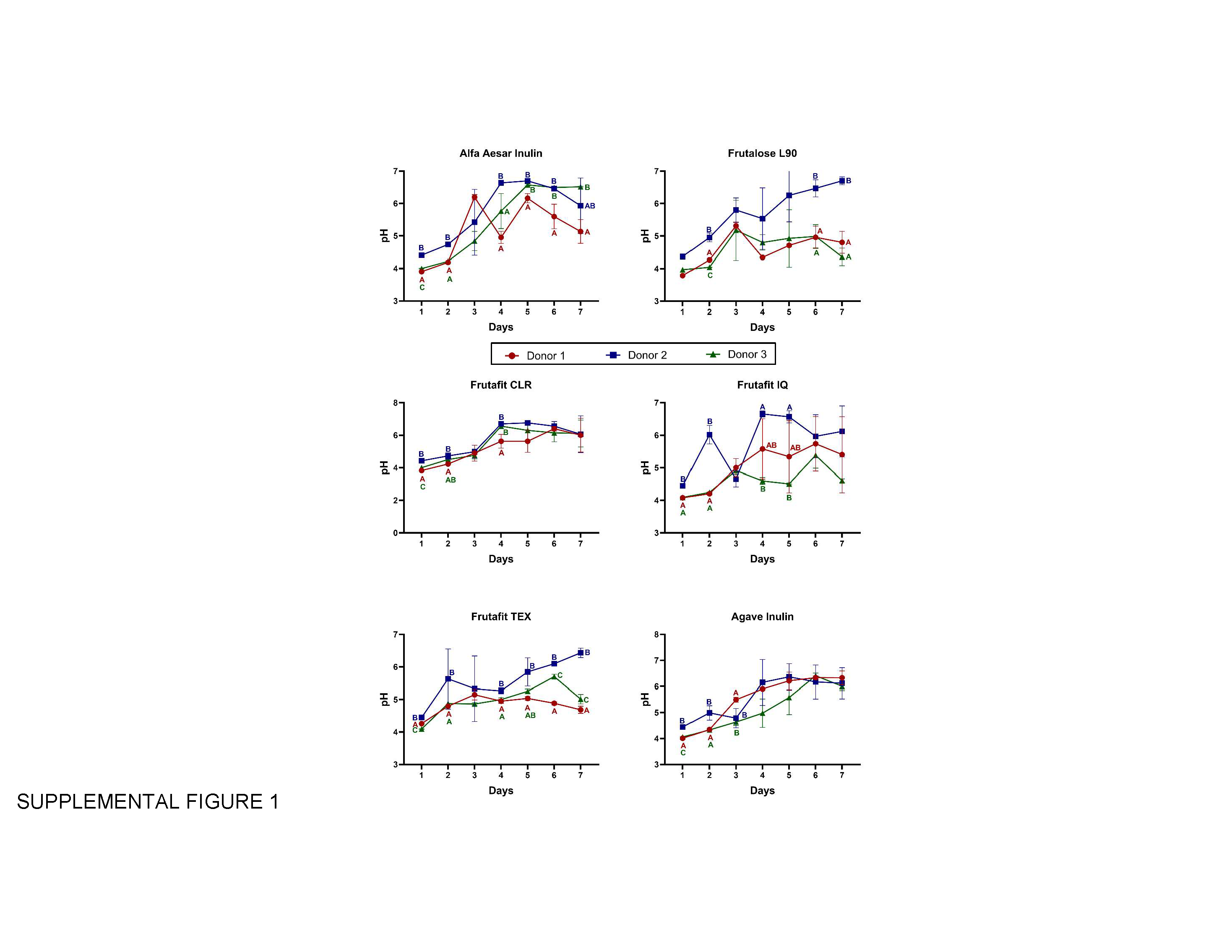

Supplement: Fig. S1 — pH changes over time. [file msystems.01031-25-s0001.tif]

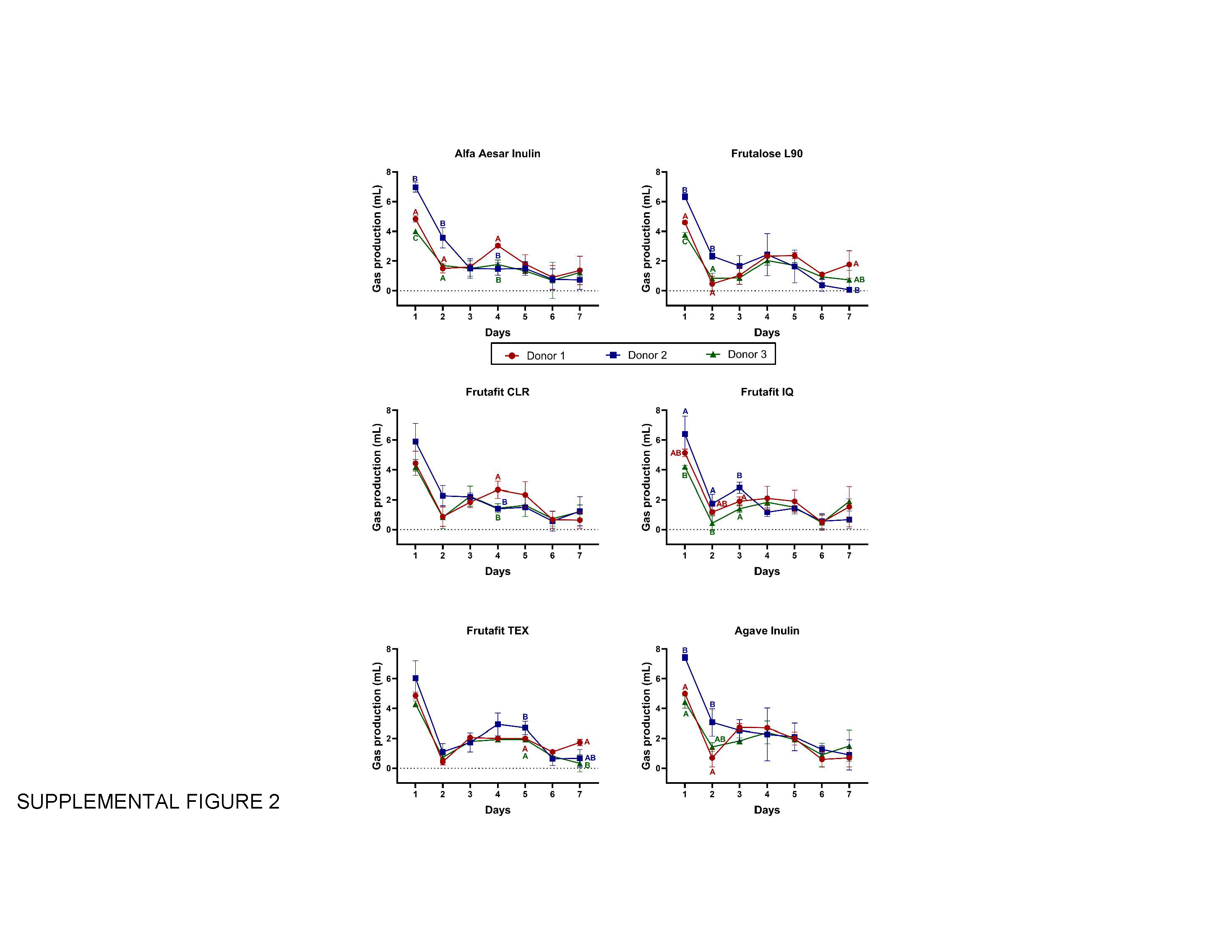

Supplement: Fig. S2 — Gas production over time. [file msystems.01031-25-s0002.tif]

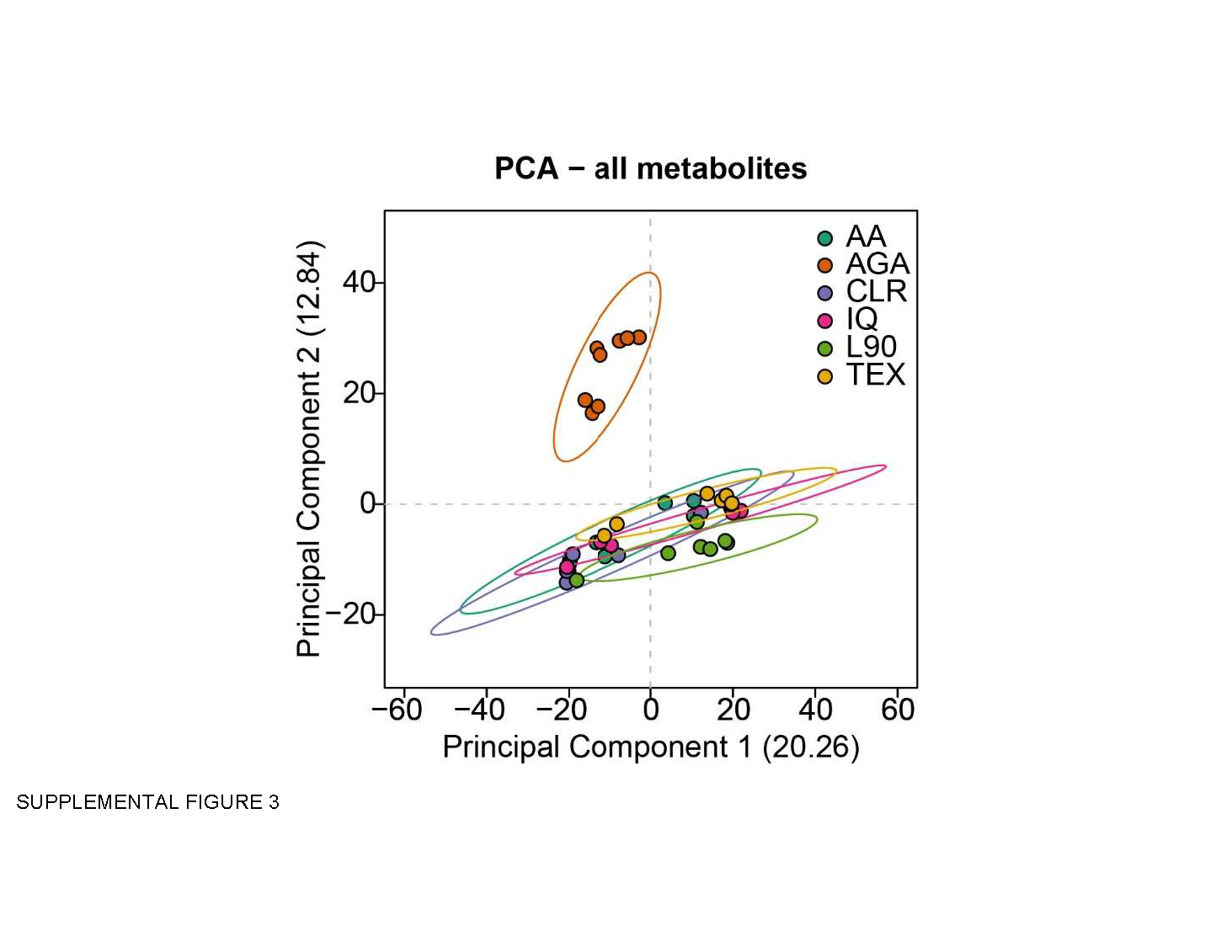

Supplement: Fig. S3 — PCA using all metabolites. [file msystems.01031-25-s0003.tif]

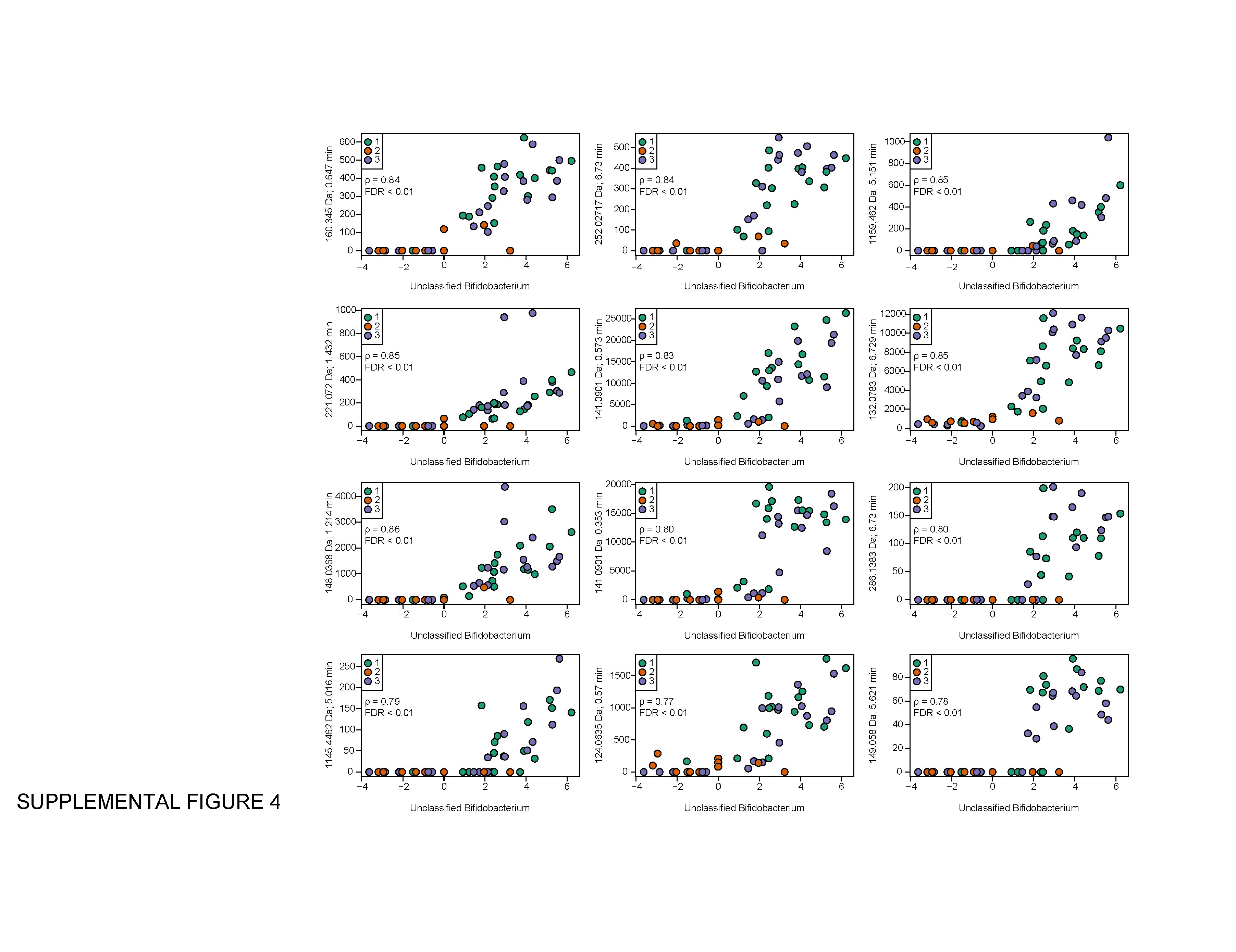

Supplement: Fig. S4 — Correlations using transformed OTUs. [file msystems.01031-25-s0004.tif]
