# Supplementary material for: Xenometabolomics reveals metabolic functional guilds unique to specific inulin subtypes in human gut microbiota cultures
Source: mSystems. 2025 Oct 22;10(11):e01031-25. doi: 10.1128/msystems.01031-25 (PMC12625710; doi:10.1128/msystems.01031-25)
Supplement: Legends — for Fig. S1 to S4 [file msystems.01031-25-s0005.docx]

**SUPPLEMENTAL FIGURES LEGENDS**

**Supplemental Figure 1. Changes in pH over 7 days of healthy adult stool cultures grown with various inulin fibers.** Abbreviations: AGA: agave inulin; AA: Alfa Aesar inulin; CLR: Frutafit CLR; IQ: Frutafit IQ; L90: Frutalose L90; Frutafit TEX.

**Supplemental Figure 2. Changes in gas production over 7 days of healthy adult stool cultures grown with various inulin fibers.** At each sampling stage, the gas overpressure of each culture was recorded by piercing the rubber stopper using a 10 mL glass syringe fitted with an 18-gauge needle. Abbreviations: AGA: agave inulin; AA: Alfa Aesar inulin; CLR: Frutafit CLR; IQ: Frutafit IQ; L90: Frutalose L90; Frutafit TEX.

**Supplemental Figure 3. Human stool culture microbial metabolite patterns differentiate inulins with varying structures.** Principal Component Analysis (PCA) scores plots including all 1219 metabolites included in analysis and considering all inulin structures. Data were log transformed and scaled to unit variance prior to analysis. Abbreviations: AGA: agave inulin; AA: Alfa Aesar inulin; CLR: Frutafit CLR; IQ: Frutafit IQ; L90: Frutalose L90; Frutafit TEX. Each inulin group consists of 2-3 lineages per individual donor (n=3), ranging between 7-8 total lineages per group.

**Supplemental Figure 4.  The top 12 Spearman’s correlations between Bifidobacterium sp. and metabolites that significantly differed in at least one binary comparison of inulins in human stool cultures.** Data for unassigned Bifidobacterium sp. operational taxonomic unit (OTU) was transformed using centered-log ratio (CLR) prior to correlation. Correlations are ranked based on the largest absolute Spearman’s correlation coefficient (ρ), and are derived from 7-8 lineages per donor, per inulin type. The circle colors indicate donor. The identities of the correlated metabolites remain unknown and thus are indicated with “ID” numbers in lieu of chemical annotations.
